# Evaluation of Efficacy and Safety of Chimeric Antigen Receptor-Natural Killer (CAR-NK) Cells in Breast Cancer: A Systematic Review and Meta-Analysis

**DOI:** 10.3390/cancers18101634

**Published:** 2026-05-19

**Authors:** Nabeel Ahmed, Jawaria Jabeen, Safa Noor, Malja Rehman, Sana Tahseen, Asmaa Qamar, Muhammad Anas, Muhammad Muneeb Khalid, Tao Li, Lechun Lyu, Zhiwei Hu

**Affiliations:** 1 Yunnan Key Laboratory of Plateau Thermal Medical Rehabilitation and Wellness, School of Rehabilitation, Kunming Medical University, Kunming 650500, China; p202405002@kmmu.edu.cn; 2 School of Pharmacy, Kunming Medical University, Kunming 650500, China; 3 College of Pharmacy, University of Sargodha, Sargodha 40100, Pakistan; jawariajabeen742@gmail.com (J.J.);; 4 Punjab University College of Pharmacy, University of the Punjab, Lahore 54000, Pakistan; 5 Cancer Biotherapy Center & Cancer Research Institute, Yunnan Cancer Hospital, The Third Affiliated Hospital of Kunming Medical University, Peking University Cancer Hospital Yunnan, Kunming 650106, China; 6 Pelotonia Institute for Immuno-Oncology Center for Antibody Therapeutics, The Arthur G. James Comprehensive Cancer Center, College of Medicine, The Ohio State University Wexner Medical Center, Columbus, OH 43210, USA

**Keywords:** breast cancer, CAR-NK, immunotherapy, natural killer cells, adoptive cell therapy, meta-analysis

## Abstract

**Background**: For almost two decades now, chimeric antigen receptor-natural killer cells (CAR-NK) have been investigated in pre-clinical breast cancer models, yet clinical evidence on efficacy remains scarce. This meta-analysis provides pooled evidence of pre-clinical CAR-NK effectiveness and safety in breast cancer and an overview of current clinical trials to support clinical translation. **Methods**: Following PRISMA guidelines and a registered protocol (PROSPERO, CRD420251131530), PubMed, Web of Science, and Scopus were searched up to 30 June 2025 for pre-clinical CAR-NK studies in breast cancer. Clinical studies were retrieved from clinicaltrials.gov and the International Clinical Trial Registry Platform (ICTRP) up to 1 March 2026. Pre-clinical studies without in vivo data or non-human CAR-NK cells were excluded. Primary outcomes were tumor burden (ratio of means, ROMs) and survival (median survival ratio, MSR). Data were analyzed in JASP™ and risk of bias (RoB) was assessed using SYRCLE’s tool. **Results**: Fourteen pre-clinical studies (38 CAR-NK treatment groups targeting EGFR, HER2, tissue factor, CD70, mesothelin, or folate receptor, with peripheral blood as the primary NK source, and a 5–10 million cell dose) and 11 early-phase clinical studies (targeting HER2, TROP2, PD-L1, MUC1, or NKG2D ligands under ongoing investigation) were included. In pooled pre-clinical analysis, CAR-NK significantly reduced tumor burden against untreated and unmodified/mock controls (ROM 0.311 [0.22–0.44] and 0.42 [0.33–0.53], *p* < 0.001, respectively). Survival was also prolonged significantly (MSR 1.47 [1.15–1.87], *p* = 0.010 vs. untreated; 1.30 [1.09–1.60], *p* = 0.007 vs. unmodified/mock NK cells). Subgroup analyses indicated improved efficacy with peripheral blood source and 5–10 M dosing. No treatment-related toxicities were reported. CAR-NK persistence was generally higher than unmodified/mock NK cells. **Discussion and Conclusions**: Significant heterogeneity was observed in ROM analysis which the multi-level meta-analysis configured as intra-study interventional variability. There was moderate RoB in pre-clinical studies. Published results from clinical trials remain limited, highlighting early stages of investigation. Overall, CAR-NK therapy demonstrated consistent pre-clinical efficacy and safety, supporting further translational and clinical evaluation in breast cancer.

## Simple Summary

Chimeric antigen receptor-natural killer (CAR-NK) cell therapy is being explored as a new approach to treat breast cancer, but its benefits and safety are still not well established in patients. This study brings together evidence from animal studies and provides an overview of the current state of ongoing early-stage clinical studies to better understand how well this approach works and whether it appears safe. The findings, supported by a detailed meta-analysis, show that this therapy consistently reduced tumor growth and improved survival in pre-clinical breast cancer models, with no obvious treatment-related toxicities reported. Ongoing clinical studies are testing similar approaches in patients but are still in the early phase, with results awaited. Overall, this research helps clarify the current state of evidence and supports further development of this therapy for breast cancer, while also guiding researchers on the most promising cell sources, targets, and treatment conditions for future studies.

## 1. Introduction

Breast cancer remains one of the most common malignancies diagnosed in women and is the second leading cause of onco-related mortality worldwide [1,2]. Despite significant advances in early detection, surgical management and systemic therapies, many patients still face recurrence, metastasis and treatment resistance [3,4]. This ongoing clinical challenge has driven the search for novel therapeutic strategies that go beyond conventional modalities. Among the emerging approaches, adoptive cell therapies, especially those employing genetically engineered immune cells, have generated intense interest [5]. In particular, the adaptation of chimeric antigen receptor (CAR) technology offers a promising route to target malignancies in a more specific and potent manner [6].

Originally developed for T cells, chimeric antigen receptor T-cell (CAR-T) therapies have achieved remarkable success in hematological malignancies, with early CD19-directed studies showing durable remissions [7]. Beginning in 2017, the Food and Drug Administration (FDA) has approved at least seven CAR-T products for various hematologic malignancies including multiple myeloma, B-cell acute lymphoblastic leukemia, follicular lymphoma, large B-cell lymphoma, mantle cell lymphoma, and diffuse large B-cell lymphoma, among other conditions [8]. However, translating this approach to solid tumors like breast cancer has proven challenging and so far the outcomes of CAR-T in solid tumors have not been encouraging [9]. Though the exact mechanisms remain unclear due to lack of clinical data, the unique tumor microenvironment (TME) of solid cancers, possessing heterogeneity, limiting infiltration, and imparting immunosuppression, appear to be the main culprit [6,7].

In this context, natural killer (NK) cells engineered to express CARs, the so-called CAR-NK cells, have emerged as another encouraging alternative. NK cells belong to the innate immune system and possess the capacity to kill tumor cells without prior sensitization and without reliance on antigen presentation via major histocompatibility complex (MHC) [5,10]. Moreover, NK cells present a favorable safety profile compared to CAR-T cells, partly because of their MHC-independent recognition, lower inherent risk of graft-versus-host disease (GvHD), and generally lower association with severe inflammatory toxicities [1,5,6,11]. Given these strengths, CAR-NK therapy presents a promising platform for solid tumors such as breast cancer.

There is a growing body of pre-clinical and clinical research evaluating CAR-NK cells in solid cancers. As of 2024, at least 40 clinical trials were registered evaluating CAR-NK in various solid cancers including ovarian cancer, pancreatic cancer, breast cancer, and gastric cancers, among others [7,12]. The number is growing as with the latest search during the conduct of this study, we identified 55 clinical trials of CAR-NK in solid tumors, 85 in hematologic malignancies, and 22 in autoimmune diseases. Despite this progress, so far, no CAR-NK product has been approved for any type of malignancy.

In the context of breast cancer (one of the most studied solid cancers with CAR-NK), apparently only three trials are currently registered (at clinicaltrials.gov and International Clinical Trial Registry Platform (ICTRP)) that focus specifically on breast cancer: IRCT20240610062069N1 (recruiting), NCT05686720 (not yet recruiting), and NCT04927884 (terminated). A few other trials (for example NCT02839954, NCT05528341 and NCT05137275) include breast cancer among broader solid tumor cohorts, but their status is variable and published clinical data remain absent. Alternatively, a number of pre-clinical studies have been published evaluating CAR-NK effectiveness in breast cancer models. Studies have employed various cell lines (for example HER2, PD-L1-expressing breast cancer models) and in vivo systems to assess cytotoxic efficacy, trafficking and tumor burden reduction [3,4]. However, despite this proliferation of data, the evidence remains scattered, heterogeneous in design and endpoints, and a comprehensive synthesis is lacking. This gap between pre-clinical promise and clinical translation represents an urgent need for a consolidated meta-analysis of pre-clinical efficacy data to inform future clinical work.

Accordingly, the purpose of this systematic review and meta-analysis is to aggregate and quantitatively summarize the available pre-clinical studies of CAR-NK therapy in breast cancer models and describe the current state of investigation in clinical trials. We provide a systematic synthesis of CAR-NK efficacy in reducing tumor burden and improving survival, compare it with the simple allogenic NK cell therapy and also provide detailed insights into safety and persistence of CAR-NK therapy in breast cancer pre-clinical models. The objective of this review is to provide consolidated translational evidence for advancing confidence in clinical investigations.

## 2. Methodology

### 2.1. Study Registration and Reporting Framework

This systematic review and meta-analysis was prospectively registered in the PROSPERO database (ID: CRD420251131530). The study design and reporting adhered to the Preferred Reporting Items for Systematic Reviews and Meta-Analyses (PRISMA) guidelines [13], and the completed PRISMA checklist is provided in Supplementary Table S1.

### 2.2. Literature Search

A comprehensive search strategy for pre-clinical studies was developed and implemented across PubMed, Scopus, and Web of Science databases for all records published up to 30 June 2025. Search strings combined both controlled vocabulary and free-text keywords associated with CAR-NK therapy (e.g., “chimeric antigen receptor NK cells,” “CAR natural killer,” “CAR-NK”). Representative search queries for each of the searched databases are listed in Supplementary Table S2.

### 2.3. Eligibility Criteria

Eligible studies were original research articles evaluating human-derived CAR-NK cells in mammalian in vivo models of breast cancer. To be included, studies had to feature an untreated, sham-treated, or non-engineered NK cell control arm. Exclusion criteria were: (i) studies without an in vivo component, (ii) non-English publications, (iii) review or commentary articles, (iv) in vitro-only experiments, and (v) studies using non-antigen-specific chimeric constructs or non-genetically modified NK cells. In vitro-only studies were excluded because the primary aim of this review was to evaluate in vivo pre-clinical efficacy and safety using outcomes such as tumor burden, survival, toxicity, and persistence. These endpoints require animal models and could not be directly pooled with in vitro cytotoxicity or mechanistic assays. Studies employing non-human CAR-NK cells or lacking appropriate controls were also excluded.

### 2.4. Screening and Study Selection

All retrieved citations were imported into Rayyan™ for systematic screening [14]. Two independent reviewer teams (MR, SN, ST; AQ, MA, MMK) evaluated titles and abstracts, and discrepancies identified by the software were resolved through discussion. When disagreements persisted, the article was advanced to full-text review. The same process was followed for full-text screening, with NA and JJ acting as the mediators for conflict resolution. No automation or AI-assisted tools were used during selection.

### 2.5. Data Extraction and Synthesis

Two reviewer groups independently extracted data using standardized templates, and any inconsistencies were resolved by consensus between authors NA and JJ. Extracted data included animal strain, sex, tumor model and route, target antigen, NK cell source, transduction and CAR design, additional genetic modifications, concurrent treatments, control type, dosing regimen, administration route, and primary and secondary outcome details (summarized in tabular form) for pre-clinical studies.

For pre-clinical studies, primary efficacy endpoints were tumor burden and survival. Tumor burden was recorded at the final time point when all animals in the intervention and the control groups were alive [15]. Tumor burden was primarily derived from bioluminescence graphs, flow cytometry, or measured tumor volume. When multiple anatomical compartments were reported, blood-derived measures were prioritized; if several quantification methods were available, data were extracted in the order of bioluminescence > flow cytometry > tumor volume > tumor weight [15]. Studies lacking quantifiable tumor data (e.g., representative images only) were excluded. Where results were presented as individual data points, mean and standard deviation (SD) values were computed for inclusion.

Survival was defined as the duration from experimental initiation to death, censoring, or endpoint, derived from Kaplan–Meier curves. If >50% of animals remained alive at study termination, that time was recorded as the median survival [16,17]. Secondary outcomes included safety indicators (toxicity, mortality, adverse effects) and CAR-NK persistence, expressed as cell counts or percentages in blood at reported time points. Data presented in graphs were digitized using PlotDigitizer™ (https://plotdigitizer.com/). Corresponding authors were contacted for missing or unclear data, but no responses were received. Experiments with unidentifiable animal numbers, unextractable data, or uncertain timepoints were excluded.

### 2.6. Statistical Analysis of Primary Outcomes

Tumor burden and survival data from pre-clinical studies were synthesized using the ratio of means (ROMs) and the median survival ratio (MSR), respectively, each expressed with 95% confidence intervals [15,18,19]. Although hazard ratios (HRs) are standard in clinical meta-analyses, their use in pre-clinical studies is limited by incomplete reporting of hazard functions and lack of individual-level data. In contrast, MSR can be reliably extracted from Kaplan–Meier curves and has been adopted in prior pre-clinical meta-analyses [15,19,20]. Additionally, methodological work has shown that MSR performs comparably to HR in small, heterogeneous experimental datasets, supporting its use as a practical alternative [18].

ROM was calculated as mean_intervention_/mean_control_, where values < 1 indicated greater efficacy of CAR-NK treatment. MSR was defined as median_intervention_/median_control_, with values >1 indicating survival benefit for CAR-NK. Median survival values were derived from digitized Kaplan–Meier curves using R survival packages.

A classical meta-analysis was performed in JASP 0.95.0.0 for ROM and MSR against true controls (untreated or sham-treated animals) and mock controls (unmodified/mock NK cells). Standard errors for ROM were based on SDs, while those for MSR were computed using standard error = 1/√*n*. The type of model (random or fixed effects) was determined based on the presence of significant heterogeneity (I^2^) and was interpreted as follows: low (0–40%), moderate (30–60%), substantial (50–90%), or considerable (75–100%). Where indicated, a multi-level meta-analysis was also employed to further explore the sources of heterogeneity. Here, three hierarchical levels were defined, i.e., study, experiment, and group, with treatment groups nested within experiments and experiments nested within studies, yielding the random-effect model: random = list(Component 1 = ~1 | Study/Exp/Group). I^2^ in this case was estimated following ref. [17,21]. Sensitivity analyses involved identifying influential estimates and recalculating pooled effects after their exclusion. Publication bias was evaluated through funnel plots and trim-and-fill adjustment in JASP™.

### 2.7. Risk-of-Bias Assessment

Risk of bias was evaluated using the SYRCLE risk-of-bias tool for animal studies [22]. Data were extracted independently by the reviewer teams as described above, and the assessment outcomes are presented in tabular form in the Supplementary Materials.

### 2.8. Protocol Deviations

The initial registered protocol was designed to systematically capture and synthesize pre-clinical evidence on CAR-NK therapy in breast cancer. However, in the present study, the clinical literature was also systematically retrieved, which was not documented in the protocol. The deviation broadens the study spectrum without affecting the core objectives.

A search for clinical trials was conducted on clinicaltrials.gov and the ICTRP up to 1 March 2026 using the keyword “CAR-NK”. For initial screening, clinical trials utilizing CAR-NK as the intervention and breast cancer as the target population were included. Screening was performed in duplicate by two independent reviewer groups (MR, SN, ST; AQ, MA, MMK), and conflicts were resolved by mutual discussion or, in case of conflict, by NA and JJ. Data on target antigen, target conditions, CAR-NK source, intervention details, disease characteristics, trial phase, date first posted, trial status, expected enrollment, trial sponsor, country, and results availability were extracted and are presented.

## 3. Results

### 3.1. Pre-Clinical Efficacy and Safety of CAR-NK in Breast Cancer

#### 3.1.1. Study Selection and Characteristics

**Figure 1:**
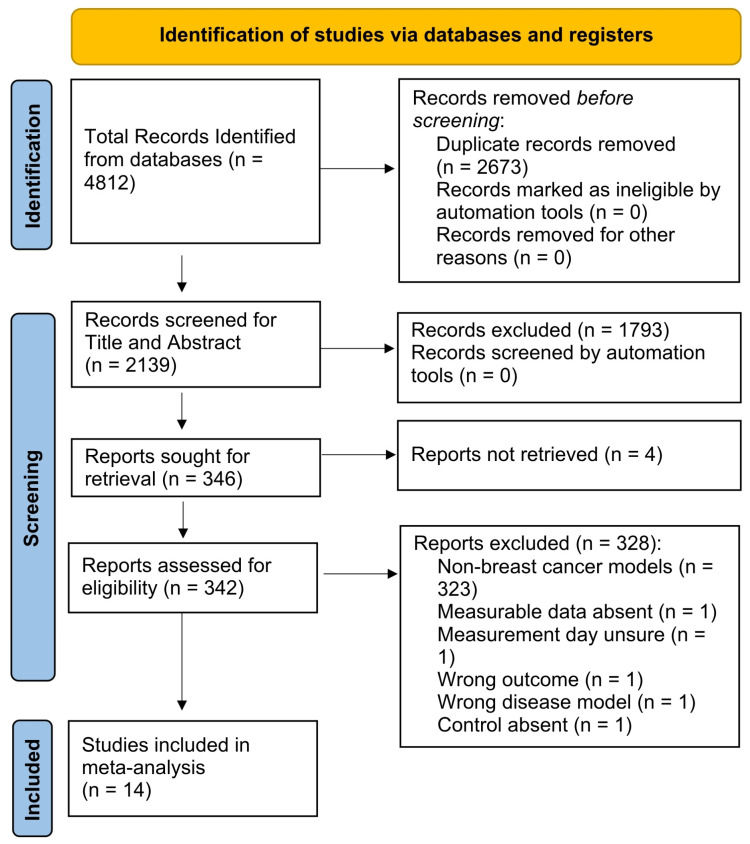
PRISMA flowchart describing the screening process for included pre-clinical studies.

The systematic search across electronic databases yielded 4812 records, of which 2673 were duplicates. After title and abstract screening of the remaining studies, 346 full-text articles were assessed for eligibility. Based on predefined inclusion and exclusion criteria, 14 pre-clinical studies were finally included in the systematic review and meta-analysis (Figure 1).

The characteristics of the included studies are summarized in Table 1. Among the included pre-clinical studies, Chen et al. [23] targeted EGFR using NK-92 cells engineered with a CD28-based CAR construct. A single intracranial dose of 2 × 10^6^ cells was administered. Kim et al. [24] also targeted EGFR, but used NK-92MI cells and an intravenous route. In this study, 5 × 10^6^ cells were given on days 7, 10, and 13. Liu et al. [25] and Jo et al. [26] further explored EGFR, albeit using PBMC-derived NK cells. The former used a 4-1BB costimulatory domain and administered the cells intravenously at 1 × 10^7^ weekly. Jo et al. [26], in contrast, used NK cells with a CD28-based CAR construct and introduced additional TIGIT-targeting modifications. CAR-NK cells were delivered intraperitoneally at 1 × 10^7^ cells on days 3 and 5.

In the pre-clinical models of Jan et al. [27], HLA-G (Human Leukocyte Antigen G) was targeted using PBMC-derived NK cells. The CAR construct included iC9, an inducible cell death receptor, and the DAP-12 signaling domain. An initial dose of 1.5 × 10^7^ cells was followed by repeated intravenous infusions of 5 × 10^6^ cells on days 7, 14, 21, and 28. Hu et al. [28] investigated tissue factor (TF) using NK-92MI cells engineered with CD28 and 4-1BB domains along with CD16 modification. Cells were administered intravenously at 3 × 10^6^ on day 0 and 2 × 10^6^ on day 17.

The second-most-targeted receptor, after EGFR, was HER (Human Epidermal Growth Factor Receptor), including HER1, HER2, and HER3. In this regard, Xia et al. [29] targeted HER2 using PBMC-derived NK cells with a CD28-based CAR construct and PD-1 extracellular domain modification. Two dosing schedules were reported based on the type of cell line used: 6 × 10^6^ cells given 7 days apart (a total of five doses), and 1 × 10^7^.

Two years apart, Gergely et al. [5] also targeted HER2 using NK-92 cells with third-generation CAR constructs and CD16 modification. A single intravenous dose of 5 × 10^6^ cells was administered on day 14. Similarly, Lee et al. [30] also administered a single intravenous dose of 5 × 10^6^ cells targeting HER3 using cord blood-derived NK cells with a 4-1BB costimulatory domain and IL-15 incorporation. The administration route also seemed to influence the dosing regimen, as in another study, Röder et al. [1] used peritumoral (instead of intravenous) delivery of HER2-NK-92 cells, but delivered 1 × 10^7^ cells (compared to 5 × 10^6^) three days apart. Further, it is worth noting that their CAR construct employed only basic components with no additional modifications, as seen in Gergely et al. (CD16) and Xia et al. (PD-1 domain). One study [11] also targeted the HER1 antigen using PBMC-derived NK cells and a CD28-based CAR construct, delivering 5 × 10^6^ cells on days 16 and 18. In this study, the authors also co-administered/implanted a puerarin-loaded polyethylene depot in tumor models, which caused vasodilation and increased HER1-CAR-NK cell infiltration into tumors.

In addition to NK-92 and PBMC-based sources, some studies also used stem cells and cord blood as NK sources. For example, Yang et al. [6] investigated MSLN (mesothelin)-targeted CAR-NK cells derived from induced pluripotent stem cells. Cells were administered intravenously on a weekly basis for seven weeks, although the exact dose was not specified. Rafei et al. [31] investigated CD70-targeted CAR-NK cells derived from cord blood, incorporating CD28 costimulation and CREM (cAMP Response Element Modulator) gene knockout. The authors demonstrated, through systematic evaluation, that CREM gene knockout in CAR-NK cells improved their function in vitro and in vivo. CAR-NK cells were administered intravenously at 3 × 10^5^ on day 7. Finally, Liu et al. [10] developed a bispecific adapter consisting of fluorescein isothiocyanate and folic acid (FITC-FA) with the ability to bind tumor folate receptor at one end and anti-FITC CAR-NK cells on the other end, potentially limiting off-target toxicities. CAR-NK cells were derived from human pluripotent stem cells with a 4-1BB domain and were delivered intravenously at 5 × 10^5^ cells on day 7.

To summarize the characteristics of included records, all studies were conducted using murine xenograft models, predominantly NSG mice (*n* = 9), followed by NOG (*n* = 2), NOD/NSGA (*n* = 1), BALB/c (*n* = 1), and other immunodeficient strains (*n* = 2). Female mice were the most frequently used subjects (*n* = 10), aligning with the relevance of estrogen receptor-positive breast cancer biology in pre-clinical modeling. For methods of transducing CAR constructs into NK cells, lentiviral transduction was the dominant engineering approach (*n* = 8, 57%), followed by retroviral systems (*n* = 4) and a single CRISPR/Cas9 modification (*n* = 1).

The costimulatory signaling domains were primarily CD28 (*n* = 6) and 4-1BB (*n* = 5), with two studies combining both [5,28] or incorporating DAP-12 [27] for enhanced activation. Approximately five studies incorporated additional functional modules, such as IL-15 signaling, the PD-1 extracellular domain, CD16 polymorphisms, or CREM gene knockout, to enhance persistence and anti-tumor efficacy. Moreover, two studies co-administered adjuvant agents to potentiate CAR-NK cytotoxicity, including doxorubicin or puerarin-loaded PEG hydrogel.

In terms of NK sources, PBMCs were most commonly employed for CAR transduction (*n* = 5, 36%), followed by NK-92 cell lines (*n* = 3), NK-92MI variants (*n* = 2), cord blood-derived NK cells (*n* = 2), and induced pluripotent stem cells (iPSCs or hPSCs; *n* = 2). The target antigens that are being investigated for CAR-NK therapy of breast cancer include EGFR (*n* = 4; [23,24,25,26]) and HER2 (*n* = 3; [1,5,29]), followed by CD19-HER3 (*n* = 1; [30]), tissue factor (*n* = 1; [28]), CD70 (*n* = 1; [31]), HLA-G (*n* = 1; [27]), mesothelin (*n* = 1; [6]), and folate receptor (*n* = 1; [10]). In summary, these studies demonstrate that most pre-clinical efforts have focused on EGFR- and HER2-directed CAR-NK constructs, using PBMC-derived or NK-92-based platforms with CD28/4-1BB costimulation.

#### 3.1.2. Experimental Details and Analysis of Primary Outcomes

The 14 included studies encompassed 38 unique CAR-NK experimental groups, representing substantial heterogeneity in tumor models, dosing regimens, and administration routes (Table 2). Most investigations utilized orthotopic (*n* = 16) or subcutaneous (SC; *n* = 16) xenografts, followed by intraperitoneal (IP), intravenous (IV), or intracranial tumor implantation (*n* = 2 each). Orthotopic models (models in which cancer cells are implanted into the same organ where the cancer originated), particularly those derived from MDA-MB-231 and HS578T human breast cancer cell lines (metastatic breast adenocarcinoma and human breast adenocarcinoma, respectively), predominated, reflecting an emphasis on mimicking the metastatic microenvironment of triple-negative breast cancer (TNBC). A smaller subset of studies employed patient-derived xenografts (PDX; *n* = 3) or murine EMT6-hHER2 lines (murine mammary adenocarcinoma) to study native tumor–immune interactions.

The CAR-NK cell dose varied widely across studies, ranging from 3 × 10^5^ to 1 × 10^7^ cells per mouse, with the majority of experiments administering doses within the 5 M–10 M range (*n* = 28, 73%). A few studies employed tiered dosing strategies such as initial higher priming doses (15 M) followed by maintenance infusions of 5 M to evaluate sustained cytotoxicity and persistence. In terms of dosing frequency, multiple-dose regimens were more prevalent (*n* = 26, 68%) than single administrations (*n* = 11, 29%). The most common schedule involved three to four injections, typically spaced at weekly intervals (e.g., days 7, 14, 21, and 28). The maximum number of doses reported was seven, corresponding to a prolonged weekly regimen over seven weeks, emphasizing long-term exposure in certain protocols.

Regarding the route of administration, IV delivery was overwhelmingly dominant (*n* = 33, 87%). Limited studies adopted intraperitoneal (*n* = 2) or intracranial (*n* = 2) routes, while one used peritumoral injection to maximize local CAR-NK infiltration. In summary, these patterns indicate a clear experimental convergence toward systemic multi-dose IV administration within the 5–10 million cell range, primarily in orthotopic xenograft settings, as the common pre-clinical model framework for assessing CAR-NK efficacy in breast cancer.

**Figure 2:**
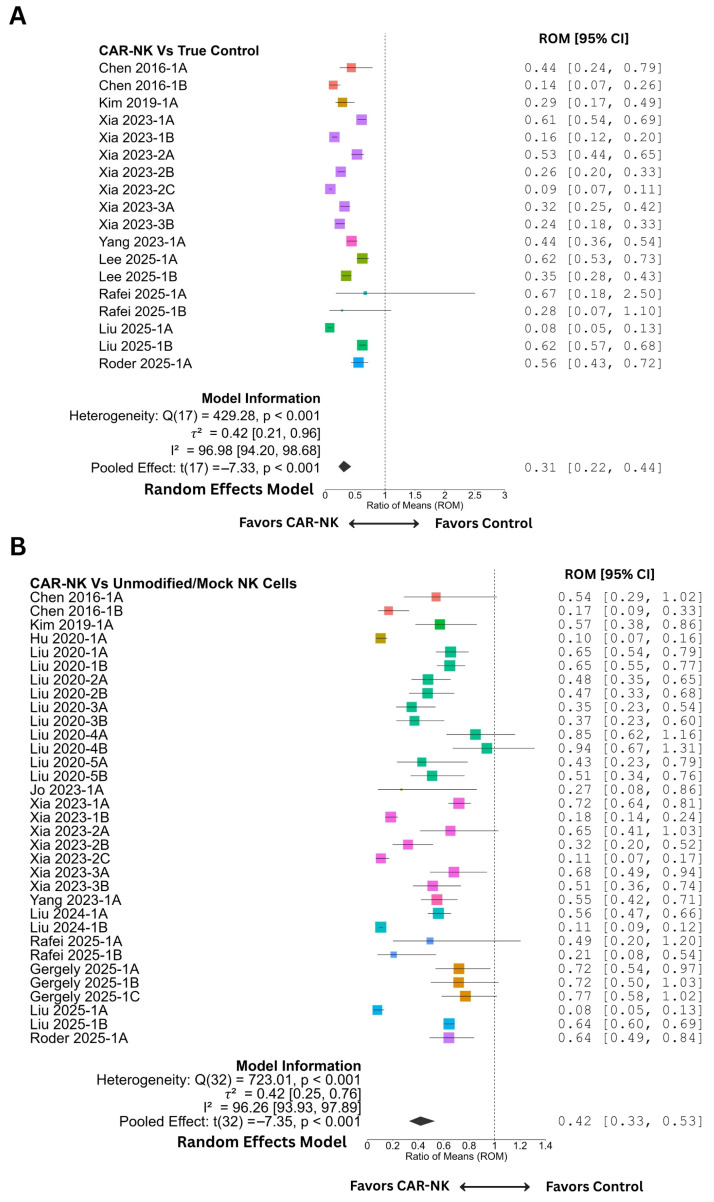
Meta-analysis of the ratio of means (ROMs) against true control and unmodified/mock NK control. (**A**) Forest plot showing individual and pooled estimates of ROM against true control (untreated or sham-treated animals). (**B**) Forest plot showing individual and pooled estimates of ROM against unmodified/mock transduced NK cell control. Individual estimates are shown as colored squares specific to each study, while the pooled estimate is shown as a black diamond at the end. A vertical line at ‘1’ on the *x*-axis indicates the null effect. Estimates less than ‘1’ (to the left) favor CAR-NK therapy. CI, confidence interval. Please refer to Table 1 and Table 2 for the corresponding references of the study IDs.

**Figure 3:**
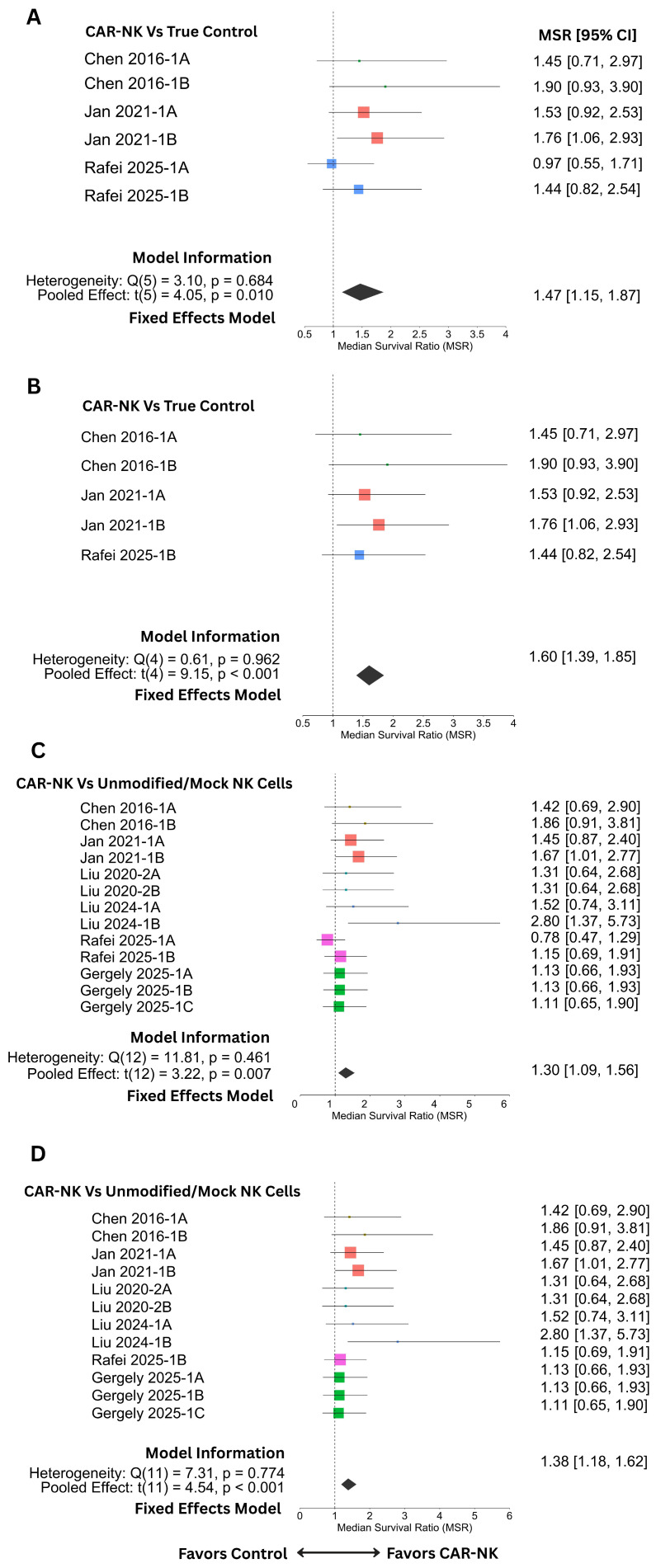
Meta-analysis of the median survival ratio (MSR) against true control and unmodified/mock NK control. (**A**) Forest plot showing individual and pooled estimates of MSR against true control (untreated or sham-treated animals). (**B**) Forest plot representing the same info as A after removing the influential estimate. (**C**) Forest plot showing individual and pooled estimates of MSR against unmodified or mock transduced NK cell control. (**D**) Forest plot showing the same information as in C after removing the influential case. Individual estimates are shown as colored squares specific to each study, while the pooled estimate is shown as a black diamond at the end. The vertical line indicates a null effect at ‘1’ on the *x*-axis. Estimates greater than ‘1’ (to the right) favor CAR-NK therapy. CI, confidence interval. Please refer to Table 1 and Table 2 for the corresponding references of the study IDs.

Next, we performed meta-analyses for primary outcomes, i.e., ROM and MSR (Figure 2 and Figure 3). The pooled analysis of tumor burden outcomes demonstrated a significant reduction in tumor volume following CAR-NK therapy compared with both untreated (true control) and unmodified/mock NK cell controls. When compared against true controls, the random-effect model was applied due to high heterogeneity among studies (Q_e_(17) = 429.28, *p* <0.001; I^2^ = 96.98%). The pooled ROM was 0.311 (95% CI: 0.222–0.435, t(17) = −7.33, *p* < 0.001), indicating that tumor volumes in CAR-NK-treated animals were reduced by approximately 69% relative to untreated controls (Figure 2A). Similarly, against the unmodified/mock NK cell control groups, a significant tumor-suppressive effect was observed (ROM = 0.417, 95% CI: 0.328–0.532, t(32) = −7.35, *p* < 0.001) (Figure 2B), again with substantial heterogeneity (Q_e_(32) = 723.01, *p* < 0.001; I^2^ = 96.26%). Sensitivity analyses (leave-one-out) revealed no influential cases, confirming the stability of pooled estimates (Supplementary Table S3).

For the survival outcomes, the meta-analysis was conducted using fixed-effect models, as no significant heterogeneity was observed in either comparison. Against true control groups, the pooled MSR was 1.468 (95% CI: 1.151–1.874, t(5) = 4.05, *p* = 0.010), suggesting that CAR-NK-treated animals exhibited an average 47% longer median survival than their untreated counterparts (Figure 3A). One influential case (Rafei 2025–1A) [31] was identified (through the leave-one-out analysis); after its removal, heterogeneity further diminished (Q_e_(4) = 0.61, *p* = 0.962), and the adjusted pooled MSR increased to 1.602 (95% CI: 1.389–1.849, t(4) = 9.15, *p* < 0.001) (Figure 3B), confirming a strong and consistent survival benefit. In comparison with unmodified/mock NK controls, CAR-NK treatment similarly conferred a significant survival advantage (MSR = 1.304, 95% CI: 1.089–1.560, t(12) = 3.22, *p* = 0.007) (Figure 3C), with no observed heterogeneity (Q_e_(12) = 11.81, *p* = 0.461). After exclusion of the same influential case (Rafei 2025-1A) [31], the pooled estimate increased to 1.384 (95% CI: 1.182–1.620, t(11) = 4.54, *p* < 0.001), confirming the robustness of findings across analytic models (Figure 3D).

In summary, these data demonstrate a consistent and statistically significant improvement in both tumor suppression and survival following CAR-NK therapy. The tumor analyses exhibited high heterogeneity, likely reflecting biological and methodological diversity, whereas survival outcomes were highly stable across studies, suggesting the reproducible survival benefits of CAR-NK treatment in pre-clinical breast cancer models.

#### 3.1.3. Investigation of Heterogeneity—Subgroup Analyses and Multi-Level Meta-Analyses

To explore heterogeneity and compare pooled estimates across specific groups, a subgroup analysis was performed, as shown in Table 3. For tumor endpoints (ROM), no statistically significant differences were observed for any subgroup analysis, while for the MSR, statistical significance was reached for ‘IL support’ and ‘dosing frequency’ variables (*p* = 0.028 and 0.011, respectively). Some key patterns that emerged from this subgroup analysis are summarized as follows: (1) peripheral blood-derived CAR-NK cells demonstrated superior performance in both efficacy endpoints, i.e., showing the lowest pooled ROM (0.266) and highest MSR (1.600), suggesting that primary PBMC-based constructs may offer more sustained cytotoxicity and in vivo persistence than immortalized NK-92 or cord blood derivatives, at least in breast tumor environments, (2) the absence of exogenous cytokine support (IL-2/IL-15) yielded more favorable results both for ROM (0.285) and MSR (1.639), indicating that intrinsic signaling through the CAR construct may sufficiently activate NK effector pathways without additional cytokine stimulation, (3) the effect of dosing frequency also aligned directionally across both endpoints, with multiple infusions providing more consistent tumor suppression and survival benefit than single administrations.

Of note, despite stratification, substantial heterogeneity in ROM estimates persisted, which could reflect variability in tumor models, implantation sites, and multi-arm study designs rather than a single modifying factor. A further investigation into heterogeneity is provided in this section using multi-level meta-analysis using the variance-partitioning approach proposed by Nakagawa and Santos (2012) [21] (3.3 of Table 3). This model decomposed variance across three hierarchical levels: (i) study, (ii) experiment within study, and (iii) treatment group within experiment. For both comparisons between CAR-NK and true control groups or mock NK control groups, virtually all the variance was concentrated at the treatment group level (σ^2^ = 0.416; I^2^ = 93.04% and σ^2^ = 0.384; I^2^ = 78.84%, respectively), while the study-level and experiment-level components were negligible (I^2^ ≈ 0% or ≤9%). This indicates that the heterogeneity initially observed in the standard random-effect model (Figure 2) primarily originated within studies, i.e., between individual treatment arms or groups rather than across independent publications. In other words, design-related or biological variations among experimental groups (e.g., dose, cell source, or tumor model) appear to be the dominant source of variability rather than methodological inconsistency between studies. This finding supports the soundness of the overall pooled effects while highlighting that future pre-clinical designs should aim for greater standardization of group-level factors (e.g., dosing, cell engineering parameters) to reduce intra-study variability.

#### 3.1.4. Synthesis on Safety and Persistence Data

We extracted descriptive data for safety and persistence measures from the included studies. Of 14 studies, only eight studies [6,10,11,25,28,29,30,31] reported data on safety aspects, while six studies did not comment [1,5,23,24,26,27] (Supplementary Table S4). Of the eight studies, five were limited to monitoring weight changes [6,11,25,28,30], while three also reported organ-level observations through tissue staining [10,29,31]. Generally, CAR-NK therapy in breast cancer pre-clinical models appeared to be well tolerated with no observable weight loss or other organ toxicities. In terms of persistence (Supplementary Table S5), four studies [1,27,29,31] commented on CAR-NK persistence in vivo in spleen, tumor (*n* = 2), and blood. CAR-NK cells were measured in these studies between 7 and 20 days post-first CAR-NK injection and were detectable. Of note, CAR-NK cells showed higher persistence in vivo compared to unmodified/mock NK cells. For example, in the study by Xia et al., the mean count of CAR-NK cells on day 14 is 173 vs. 78 for control NK cells [29]. Similarly, in the study by Rafei et al., control NK cells are negligibly detectable (0 or 0.1 cells/ul) on day 20 vs. higher counts of CAR-NK on the same day [31]. Similarly, in Röder et al., at day 11, CAR-NK cells were detected in the tumor environment, while control NK cells were absent [1]. Of note, no study reported the day CAR-NK diminished from the in vivo system.

#### 3.1.5. Risk-of-Bias Assessment

Risk of bias was evaluated for all 14 included studies using SYRCLE’s risk-of-bias tool for animal experiments (Supplementary Table S6). Overall, the methodological quality was moderate, with most studies exhibiting unclear risk in several domains due to incomplete reporting. Sequence generation and allocation concealment were seldom described; only [31] reported explicit randomization procedures, whereas studies such as [1,5,23] lacked details, indicating a potential selection bias. Baseline characteristics were generally balanced across groups (e.g., [25,26]); however, random housing and investigator blinding were rarely applied, with consistent “unclear” or “no” ratings across most studies (e.g., [10,28,29]). Similarly, blinding and randomization of outcome assessment were frequently unclear; however, for the survival endpoint, these domains were deemed not applicable, as mortality-based outcomes are objective and minimally influenced by assessor expectations. In contrast, tumor measurements were more susceptible to bias, yet only [25,31] reported any blinding procedures. Attrition and selective reporting risks were generally low, with most studies explicitly presenting complete datasets. No evidence of other major biases was identified. Collectively, these findings highlight a moderate overall risk of bias, mainly due to inadequate reporting of randomization and blinding procedures, an aspect that should be improved in future pre-clinical CAR-NK research to strengthen reproducibility and reliability.

Publication bias was assessed using funnel plots and asymmetry tests. Visual examination of the funnel plots for both tumor (ROM) and survival (MSR) outcomes suggested an overall pattern of approximate symmetry (Supplementary Figure S1). For the ROM analyses, the weighted regression test (Egger’s test) indicated mild asymmetry in the true control comparison (t(16) = −2.84, *p* = 0.012), while the meta-regression test did not support a significant trend (*p* = 0.520) (Supplementary Figure S1A). A similar borderline pattern was noted for the mock control analysis (*p* = 0.070) (Supplementary Figure S1B). Nonetheless, trim-and-fill procedures detected no missing studies in either case, implying that any publication bias, if present, is likely minimal. In the survival analyses (Supplementary Figure S1C,D), both funnel plots appeared largely symmetrical, with non-significant asymmetry tests (*p* = 0.90 for true control; *p* ≈ 0.07 for mock control). The trim-and-fill method imputed only a few potentially missing estimates (two for the true control and four for the mock control), resulting in negligible shifts in effect size (Supplementary Table S7). Overall, while slight asymmetry may exist, particularly in the tumor dataset, the evidence for systematic publication bias remains limited, and its influence on the pooled results appears unlikely to be substantial.

### 3.2. Clinical Studies of CAR-NK in Breast Cancer

**Figure 4:**
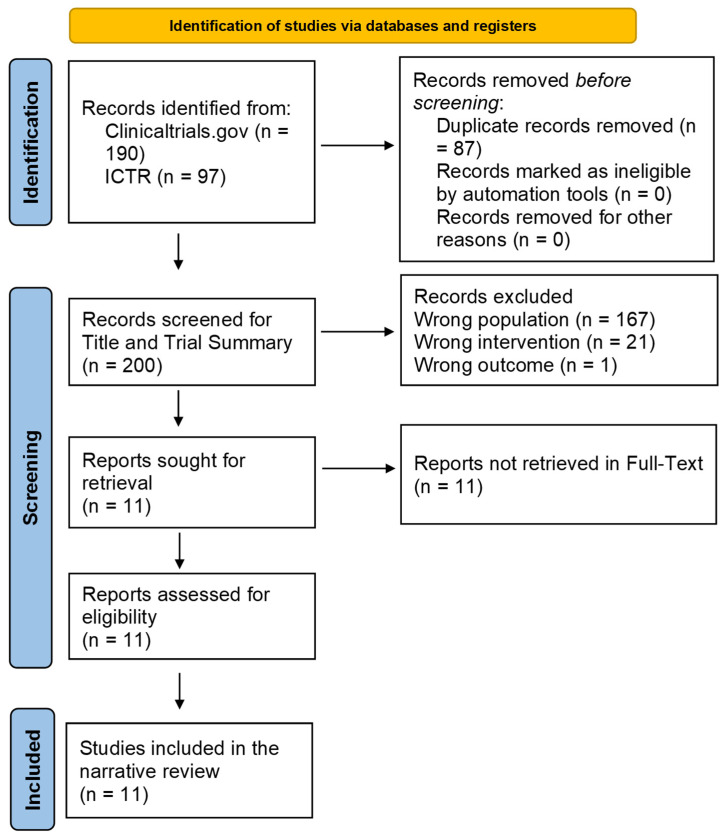
PRISMA flowchart of the search and screening process of CAR-NK clinical trials in breast cancer.

For the clinical search, a total of 287 records were retrieved from clinicaltrials.gov and WHO ICTRP (Figure 4). After excluding 181 records (*n* = 167 (wrong population/disease), *n* = 21 (wrong intervention), and *n* = 1 (wrong outcome), 11 clinical trials were included in the data synthesis (Table 4). Among the included clinical studies, NCT07410494 is investigating CAR-NK therapy targeting multiple tumor-associated antigens across a wide range of solid and hematological malignancies using donor-derived peripheral blood (PB) NK cells. The trial will employ both single- and dual-target CAR constructs, with lymphodepleting conditioning using fludarabine and cyclophosphamide prior to infusion; however, specific CAR construct details and dosing regimens were not disclosed. Similarly, NCT07410676 appears to evaluate EBNK-001, an allogeneic CAR-NK product combined with IL-15 and pembrolizumab, at escalating doses (1 × 10^8^ to 9 × 10^8^ cells) in weekly infusions (days 1, 8, and 15 per cycle), although target antigens and CAR construct details were not specified.

Several trials are focusing on defined tumor antigens. The IRCT20240610062069N1 trial is using EGFRvIII (epidermal growth factor receptor variant III) as the CAR-NK target in metastatic breast cancer from patients in Iran. The source of CAR-NK remains undisclosed, while the primary objective is to determine the dose-limiting toxicities. The regimen consists of lymphodepleting therapy with cyclophosphamide and fludarabine followed by CAR-NK cells in 1, 10, or 100 million cells/kg.

The NCT06066424 trial will target TROP2 in patients with non-small cell lung cancer (NSCLC) and breast cancer using cord blood-derived CAR-NK cells, combined with lymphodepleting chemotherapy and rimiducid; however, dosing details and CAR construct specifications were not reported. NCT05528341 is targeting NKG2D ligands using NK-92 cells, administering CAR-NK cells intravenously at 0.5 × 10^6^–2 × 10^6^ cells/kg twice weekly, representing one of the more clearly defined dosing strategies. Likewise, NCT05678205 is evaluating HER2-targeted CAR-NK cells derived from cord blood that incorporate OX40L, CD28, and IL-15 into the CAR construct, although the dosing frequency was not specified.

Other antigen-specific approaches included MICA/B (NCT05395052), PD-L1 (NCT04927884), 5T4 (NCT05137275), and MUC1 (NCT02839954), using a range of NK cell sources, including induced pluripotent stem cells (iPSCs), NK-92 cells, and unspecified allogeneic NK platforms. Briefly, the PD-L1-targeted trial (NCT04927884) has specified a dosing regimen of 2 × 10^9^ cells intravenously on days 1 and 8 of a 3-week cycle, whereas the 5T4-targeted study (NCT05137275) uses a dose-escalation design ranging from 3 × 10^9^ to 9 × 10^9^ cells, with repeated infusions within each cycle. In contrast, several studies, including NCT05686720 and NCT02839954, did not disclose detailed CAR construct characteristics despite implementing dose-escalation strategies or repeated infusion schedules.

To summarize the clinical landscape, breast cancer is most commonly included within basket trials enrolling multiple solid tumors, although three studies (NCT05686720, NCT04927884, IRCT20240610062069N1) specifically target breast cancer, and two other trials (NCT05137275, NCT02839954) focus on TNBC among other malignancies (Table 4). Moreover, a range of tumor-associated antigens is being targeted, including EGFRvIII, HER2, TROP2, PD-L1, MUC1, NKG2D ligands (e.g., MICA/B), and 5T4, as well as several undisclosed targets.

**Figure 5:**
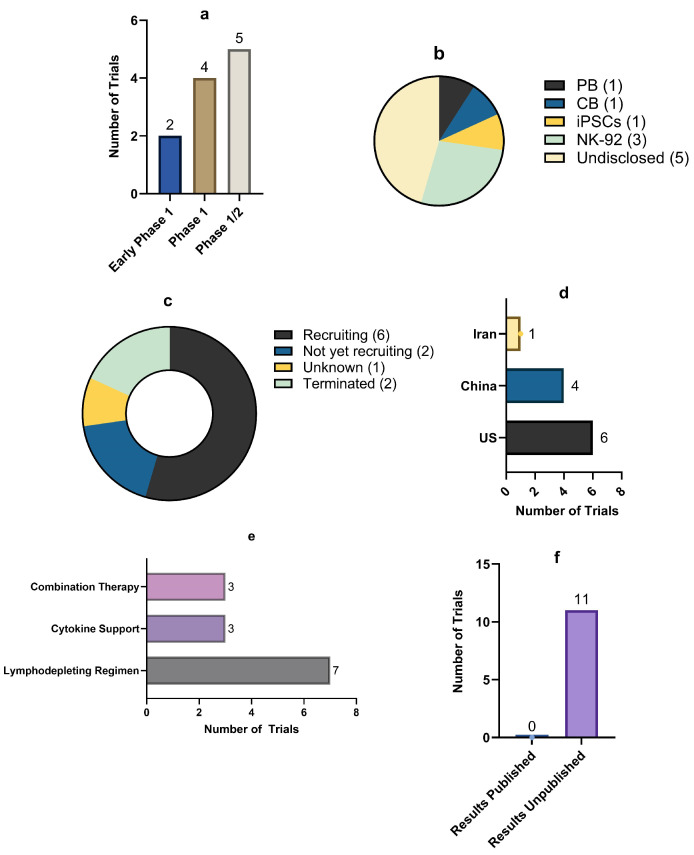
Summary statistics of CAR-NK trials in breast cancer. Summary of (**a**) trial phases, (**b**) NK-sources, (**c**) trial status, (**d**) trial country, (**e**) intervention characteristics, and (**f**) publication of results.

Figure 5 provides the numerical summary of the included clinical trials of CAR-NK in breast cancer. As shown, all studies are early-phase, including phase 1 (*n* = 4), phase 1/2 (*n* = 5), and early-phase 1 trials (*n* = 2), suggesting that clinical translation remains at an early stage, with a predominant focus on safety, feasibility, and dose escalation rather than confirmatory efficacy in breast cancer. In parallel, diverse NK cell sources are employed, including PB-derived NK cells (*n* = 1), cord blood-derived NK cells (*n* = 1), and induced pluripotent stem cell (iPSC)-derived NK cells (*n* = 1), with NK-92 cell lines being the most common (*n* = 3 trials).

Geographically, the trials are primarily being conducted in the United States (*n* = 6), China (*n* = 4), and Iran (*n* = 1), involving both academic institutions and biotechnology companies (Table 4; Figure 5). Most studies are currently recruiting (*n* = 6) or not yet recruiting (*n* = 2), while two have been terminated and one has an unknown status. Importantly, publicly available results remain extremely limited; only one trial (NCT02839954) reports results, and these do not include breast cancer-specific outcomes (Table 4). Overall, the clinical landscape suggests that CAR-NK therapy for breast cancer is still in an early developmental phase. Although multiple targets and cellular platforms are under active investigation, clinical efficacy data specific to breast cancer are not yet available, highlighting the current gap between promising pre-clinical evidence and validated clinical benefit.

## 4. Conclusions and Perspective

This systematic review and meta-analysis aimed to provide consolidated evidence to support the clinical translation of CAR-NK therapy in breast cancer. Though the pre-clinical research for CAR-NK in breast cancer has been ongoing for more than a decade now, the clinical outcomes remain yet to be determined. In this meta-analysis, we systematically show, based on 14 pre-clinical studies comprising 38 CAR-NK experimental groups, that CAR-NK therapy reduces tumor burden and prolongs median survival. We have also shown that CAR-NK therapy outperforms simple allogeneic NK cell therapy across both tumor and survival outcomes, further highlighting the benefit of adding the ‘CAR’ modification to simple NK cells. Furthermore, no major toxicities were observed in any of the CAR-treated experimental groups included in our systematic review, providing an essential advantage over CAR-T therapies, which are known to be associated with adverse effects such as GvHD or cytokine-release syndrome (CRS).

There is a scarcity of published literature on pre-clinical and clinical meta-analyses in the context of CAR-NK therapy. A recent meta-analysis provided insights into the pre-clinical efficacy of CAR-NK in hematologic malignancies, where similar findings emerged as CAR-NK therapy showed statistically significant overall benefit in improving tumor and survival outcomes [15]. These pre-clinical findings align with broader hematologic clinical trial literature on CAR-NK therapy, where encouraging response rates have been observed [12,32], therefore validating the pre-clinical consolidated evidence. Though solid cancers, such as breast cancer, have more suppressive environments, a similar direction of outcomes is expected in clinics, aligning with the outcomes of this meta-analysis.

We also observed some statistically significant differences favoring the absence of IL support and higher dosing regimens. Cytokines such as IL-15 and IL-2 are commonly employed in CAR-NK therapy to support survival, cytotoxicity, and persistence of NK cells in vivo [12,33]. Two common methods of IL support include co-administration or incorporation of the IL into the CAR construct. The former has been associated with toxicities in pre-clinical models [34]. Bastin et al. also attributed the non-significant improvement in survival in IL-15 subgroups to toxicities associated with this cytokine [15]. In the present case, the IL subgroup comprised both types of supporting mechanisms, i.e., co-administration and incorporation, as stratification would have reduced the number of observations in this subgroup. Nonetheless, in the safety outcomes, these studies did not report any potential toxicities, suggesting that these analyses may have been confounded by other predictors. For example, in Lee 2025-1B [30] (Table 2), we observe a very low ROM (0.3508) when IL-15 is incorporated into the CAR construct, whereas the opposite is observed in Rafei 2025-1A [31], where ROM ranges from 0.5 to 0.7, and MSR is less than one. Of note, in the latter study, the target antigen is CD70, unlike HER3 in Lee 2025-1B [30], which may explain the high ROMs and low MSRs, as CD70 is not a widely studied antigen in breast tumors.

Another significant difference was observed in dosing strategies: single doses or doses less than 5 M were not statistically associated with improved median survival. This suggests that in solid tumor environments, a higher or multiple-dose strategy may be more beneficial than alternatives. The clinical trial literature on CAR-NK also suggests a similar kind of observation; for example, in a recent clinical review, it was observed that the flat dosing range of CAR-NK in hematologic malignancies was 100 thousand–2 billion cells, while that for solid cancers was 1 million–3 billion cells [12], highlighting that, indeed, dosing in solid cancers may require a more aggressive and potent cell product to achieve desired efficacy outcomes.

The NK cell source is an important translational variable, although our subgroup findings should be interpreted as exploratory rather than confirmatory. In the present analysis, PB-derived CAR-NK cells showed favorable efficacy trends across tumor and survival endpoints, but subgroup differences for ROM did not reach statistical significance, limiting conclusions regarding the superiority of one source. This is consistent with Bastin et al., who reported no statistically significant difference between CAR-NK cell sources in hematologic pre-clinical models, despite a trend toward improved tumor reduction with iPSC-derived CAR-NK cells and overlapping confidence intervals [15]. From a clinical translation standpoint, PB-derived NK cells may retain greater physiological relevance but face donor variability and manufacturing challenges, whereas NK-92/NK-92MI platforms provide a more standardized and expandable product but usually require irradiation, which may limit in vivo persistence [12,15]. Stem-cell-derived CAR-NK products may offer scalable “off-the-shelf” manufacturing, but their relative advantage in solid tumors remains unproven. In ovarian cancer models, Ahmed et al. similarly reported no statistically significant subgroup differences by cell source, despite trends favoring NK-92-based CAR-NK cells, reinforcing that source-related comparisons remain hypothesis-generating [17]. Clinically, at least 40 CAR-NK trials in solid cancers have been registered, but published solid tumor outcomes remain limited and do not yet establish a clearly superior NK source; one early-phase NSCLC study used dual-targeted MUC-1/PD-L1 CAR-NK92MI cells and reported feasibility and safety in a small cohort, but this does not allow cross-source comparison [12]. Therefore, future pre-clinical and clinical studies should directly compare NK sources under otherwise matched CAR design, dose, and tumor model conditions.

An important consideration in the pre-clinical meta-analysis was substantial heterogeneity in tumor burden (ROM) analyses, which persisted even after subgroup stratification. Although multi-level meta-analysis demonstrated that this variability was primarily driven by within-study differences across treatment groups rather than between-study inconsistency, the presence of such heterogeneity still limits the precision and generalizability of pooled estimates. This reflects the inherent diversity of pre-clinical experimental designs, including differences in tumor models, dosing strategies, and CAR-NK constructs. Therefore, the pooled ROM results should be interpreted as indicative of overall trends in treatment efficacy rather than precise quantitative effect sizes, and future studies would benefit from greater standardization of experimental parameters.

Furthermore, TME-related barriers are highly relevant to CAR-NK efficacy in solid tumors, though these mechanisms could not be formally incorporated into the quantitative analysis because they were not consistently reported across included studies. Factors such as antigen heterogeneity, impaired immune-cell trafficking, stromal exclusion, hypoxia, suppressive cytokines, and checkpoint-mediated inhibition may influence CAR-NK activity in breast cancer models and could partly explain variability in treatment effects. In this regard, the TF-targeting CAR-NK approach could be promising for overcoming tumor microenvironment barriers, as tissue factor (TF) is not only overexpressed by breast cancer cells and breast cancer stem cells but is also specifically expressed by tumor vascular endothelial cells in patients’ breast tumors [28]. Nevertheless, future pre-clinical studies should include standardized reporting of TME-related parameters to enable mechanistic subgroup analyses and improve translational interpretation.

The clinical literature of CAR-NK in breast cancer is still in its early phases. We retrieved 11 such clinical trials, of which two have been terminated, and the others are still ongoing. Published results, however, are not available at the moment. Comparatively, Park et al. recently conducted a meta-analysis of ‘*natural killer*’ cell therapy in solid cancers, including breast cancer [35]. We have extracted the relevant breast cancer clinical data from the study (Supplementary Table S8) and observed the following outcomes: complete response (2.85%), partial response (16.19%), stable disease (49.5%), and progressive disease (31.4%) in 105 patients. The data suggest that while objective responses remain relatively limited, a considerable proportion of patients achieve disease stabilization following NK cell-based therapy. This pattern indicates potential anti-tumor activity and disease-controlling effects of NK cell immunotherapy in breast cancer. Nevertheless, these findings largely reflect conventional NK cell-based approaches rather than CAR-engineered platforms, and therefore cannot be directly extrapolated to CAR-NK therapy. As summarized in Table 4, ongoing clinical trials will be essential to determine whether CAR modification can further enhance the therapeutic efficacy and durability of NK cell-mediated responses in breast cancer.

Despite the encouraging conclusions, we acknowledge several limitations in our study. First, we assessed tumor burden at the last time point when all animals in each experiment were still alive, as reported previously [15,17]. While this approach ensures we compare groups at the same time, it can lead to an underestimate of therapeutic benefit. Additionally, the risk of bias within the included studies is an important consideration. Many studies did not clearly report methods to minimize bias—for example, random allocation of mice to treatment vs. control or blinding of outcome assessors was often not stated. This highlights the need for future studies to follow established protocols, such as SYRCLE’s risk-of-bias tool, to instill greater confidence in therapeutic outcomes. Finally, the subgroup comparisons in our analysis were pooled from separate studies rather than compared directly, and they remain exploratory and may be affected by confounding.

In conclusion, this SRMA underlines the potential anti-tumor capabilities of CAR-NK cells in breast cancer models and provides valuable insights for clinical translation. The findings from this study, though exploratory and hypothesis-generating, support the conclusion that there may be an overall benefit to using CAR-NK in breast cancer and that CAR-NK may be more effective than naïve NK cells. Furthermore, the findings support a favorable safety profile of CAR-NK cells, which is equally important for clinical translation. The current evidence is largely pre-clinical or at the initial stages of clinical investigation, highlighting the need for further clinical development. Additionally, the study highlights several translational aspects for future investigations, including direct comparisons of engineering strategies, compliance with established reporting standards, evaluation of persistence outcomes, and inclusion of TME variables in pre-clinical models.

## Figures and Tables

**Table 1:** Key descriptions of included studies.

No.	Study	Population				Intervention				Reference
		Animal Strain	Sex	CAR Target	Cell Source	Transduction Method	Costimulatory Domains	Additional Modifications to CAR Cells	Co-Administered Treatments	
1	Chen 2016	NSG	ns	EGFR	NK-92 Cells	Lentivirus	CD28	-	-	[23]
2	Kim 2019	NOG	ns	EGFR	NK-92MI Cells	Multifunctional Nanoparticles	-	-	-	[24]
3	Jan 2021	NSG	Female	HLA-G	PBMCs	Lentivirus	ns	iC9, DAP-12	Doxorubicin, IL-15, IL-2	[27]
4	Hu 2020	NSG	Female	Tissue Factor	NK-92MI Cells	Lentivirus	CD28, 4-1BB	CD16	-	[28]
5	Liu 2020	Nude	Female	EGFR	PBMCs	Lentivirus	4-1BB	-	-	[25]
6	Jo 2023	NSG	Female	EGFR	PBMCs	Retrovirus	CD28	No, but TIGIT targeting RNP-loaded RPs with CAR transgene	-	[26]
7	Xia 2023	NOG, BALB/c	Female	HER2	PBMCs	Lentivirus	CD28	PD-1 extracellular domain	-	[29]
8	Yang 2023	B-NDG	ns	MSLN	iPSCs	Lentivirus	4-1BB	-	-	[6]
9	Lee 2025	NOD (NSGA)	Female	CD19 and HER3	Cord blood	Lentivirus	4-1BB	IL-15	-	[30]
10	Liu 2024	NSG	ns	HER1	PBMCs	Lentivirus	CD28	-	Puerarin-loaded PEG hydrogel	[11]
11	Rafei 2025	NSG	Female	CD70	Cord Blood	Retrovirus	CD28	CREM gene knockout, IL-15 incorporation	-	[31]
12	Gergely 2025	NSG	Female	HER2	NK-92 Cells	Retrovirus	CD28, 4-1BB	CD16 (FcγRIIIA 176V)	IL-2	[5]
13	Liu 2025	NSG	Female	FITC Folate	hPSCs	CRISPR/Cas9	4-1BB	-	CAR hPSC-neutrophils, FITC-FA bispecific adapter	[10]
14	Röder 2025	NSG	Female	HER2	NK-92 Cells	Lentivirus	CD28	-	-	[1]

**ns**, not specified; **NSG**, Non-Obese Diabetic (NOD) Severe Combined Immunodeficiency (SCID) Gamma mice; **NOG**, NOD/Shi-sc/IL-2Rγnull mice; **BALB/c**, Bagg Albino/c mice; **B-NDG**, NOD-*Prkdc*em26Cd52 *Il2rg*em26Cd22/Nju mice (all immunodeficient mouse models); **EGFR**, Epidermal Growth Factor Receptor; **HLA-G**, Human Leukocyte Antigen G; **HER1/HER2/HER3**, Human Epidermal Growth Factor Receptor 1/2/3; **PBMCs**, Peripheral Blood Mononuclear Cells; **iPSCs**, Induced Pluripotent Stem Cells; **hPSCs**, Human Pluripotent Stem Cells; **CRISPR/Cas9**, Clustered Regularly Interspaced Short Palindromic Repeats/CRISPR-Associated Protein 9; **iC9**, Inducible Caspase 9; **DAP-12**, DNAX Activation Protein of 12 kDa; **TIGIT**, T-cell Immunoreceptor with Ig and ITIM domains; **RNP**, Ribonucleoprotein; **RPs**, Retroviral particles; **PD-1**, Programmed Cell Death Protein 1; **IL-2/IL-15**, Interleukin-2/Interleukin-15; **CREM**, cAMP Response Element Modulator; **CD16 (FcγRIIIA 176V)**, Cluster of Differentiation 16 (Fc gamma Receptor IIIA 176V); and **FITC-FA**, Fluorescein Isothiocyanate—Folic Acid.

**Table 2:** Experimental details from individual treatment groups with corresponding estimates of tumor and survival outcomes.

Study	Exp No.	Group ID	Pre-Tx (Mice)	Tumor Model	Tumor Route	Disease Description	Target Antigen	Additional Modifications	Concurrent Treatments	CAR-NK Dose	Dosing Day	CAR-NK Route	Control	Tumor Burden Measurement				Survival Measurement	
														Day Measured	Method	ROM_1	ROM_2	MSR_1	MSR_2
Chen 2016 [23]	1	A	N	MDA-MB-231 Cells	Intracranial	Metastatic Breast Adenocarcinoma	EGFR	-	-	2 × 10^6^	10	Intracranial	Vehicle	18	Biol	0.4370	0.5407	1.4524	1.4186
Chen 2016 [23]	1	B	N	MDA-MB-231 Cells	Intracranial	Metastatic Breast Adenocarcinoma	EGFR	-	oHSV-1, 2.00 × 10^5^ plaque-forming units on day 15	2 × 10^6^	10	Intracranial	Vehicle	18	Biol	0.1352	0.1673	1.9048	1.8605
Kim 2019 [24]	1	A	N	MDA-MB-231 Cells	Orthotopic	Metastatic Breast Adenocarcinoma	EGFR	-	-	5 × 10^6^	7, 10, 13	IV	DPBS	21	Tumor Volume	0.2899	0.5700	-	-
Jan 2021 [27]	1	A	N	MDA-MB-231 Cells	Orthotopic	Metastatic Breast Adenocarcinoma	HLA-G	iC9, DAP-12	IL-15, 10 ng/mouse, daily IL-2, 10,000 U/mouse, q2d	1.5 × 10^7^ (Day 7), then 5 × 10^6^	7, 14, 21 and 28	IV	Vehicle	-	-	-	-	1.5278	1.4474
Jan 2021 [27]	1	B	N	MDA-MB-231 Cells	Orthotopic	Metastatic Breast Adenocarcinoma	HLA-G	iC9, DAP-12	Doxorubicin, 0.5 mg/kg, for 4 weeks IL-15, 10 ng/mouse, daily IL-2, 10,000 U/mouse, q2d	1.5 × 10^7^ (Day 7), then 5 × 10^6^	7, 14, 21 and 28	IV	Vehicle	-	-	-	-	1.7639	1.6711
Hu 2020 [28]	1	A	N	PDX	Orthotopic	Invasive Ductal Breast Carcinoma	Tissue Factor	-	-	3 × 10^6^ on Day 0, then 2 × 10^6^ on Day 17	ns	IV	Unmodified NK Cells	22	Tumor Volume	-	0.1045	-	-
Liu 2020 [25]	1	A	N	HS578T Cells	Orthotopic	Human Breast Adenocarcinoma	EGFR	-	-	1 × 10^7^	14, 21, 28 and 35	IV	Mock CAR-NK Cells	28	Tumor Volume	-	0.6546	-	-
Liu 2020 [25]	1	B	N	HS578T Cells	Orthotopic	Human Breast Adenocarcinoma	EGFR	No, but different ScFv sequence	-	1 × 10^7^	14, 21, 28 and 35	IV	Mock CAR-NK Cells	28	Tumor Volume	-	0.6480	-	-
Liu 2020 [25]	2	A	N	MDA-MB-468 Cells	Orthotopic	Metastatic Breast Adenocarcinoma	EGFR	-	-	1 × 10^7^	14, 21, 28 and 35	IV	Mock CAR-NK Cells	28	Tumor Volume	-	0.4770	-	1.3125
Liu 2020 [25]	2	B	N	MDA-MB-468 Cells	Orthotopic	Metastatic Breast Adenocarcinoma	EGFR	No, but different ScFv sequence	-	1 × 10^7^	14, 21, 28 and 35	IV	Mock CAR-NK Cells	28	Tumor Volume	-	0.4735	-	1.3125
Liu 2020 [25]	3	A	N	MDA-MB-231 Cells	Orthotopic	Metastatic Breast Adenocarcinoma	EGFR	-	-	1 × 10^7^	14, 21, 28 and 35	IV	Mock CAR-NK Cells	28	Tumor Volume	-	0.3483	-	-
Liu 2020 [25]	3	B	N	MDA-MB-231 Cells	Orthotopic	Metastatic Breast Adenocarcinoma	EGFR	No, but different ScFv sequence	-	1 × 10^7^	14, 21, 28 and 35	IV	Mock CAR-NK Cells	28	Tumor Volume	-	0.3708	-	-
Liu 2020 [25]	4	A	N	MCF7 Cells	Orthotopic	Metastatic Breast Adenocarcinoma	EGFR	-	-	1 × 10^7^	14, 21, 28 and 35	IV	Mock CAR-NK Cells	28	Tumor Volume	-	0.8498	-	-
Liu 2020 [25]	4	B	N	MCF7 Cells	Orthotopic	Metastatic Breast Adenocarcinoma	EGFR	No, but different ScFv sequence	-	1 × 10^7^	14, 21, 28 and 35	IV	Mock CAR-NK Cells	28	Tumor Volume	-	0.9390	-	-
Liu 2020 [25]	5	A	N	PDX	Orthotopic	-	EGFR	-	-	1 × 10^7^	7, 14, and 21	IV	Mock CAR-NK Cells	28	Tumor Volume	-	0.4286	-	-
Liu 2020 [25]	5	B	N	PDX	Orthotopic	-	EGFR	No, but different ScFv sequence	-	1 × 10^7^	7, 14, and 21	IV	Mock CAR-NK Cells	28	Tumor Volume	-	0.5092	-	-
Jo 2023 [26]	1	A	N	MDA-MB-231 Cells	IP	Metastatic Breast Adenocarcinoma	EGFR	-	-	1 × 10^7^	3, 5	IP	Mock CAR-NK	26	Biol	-	0.2679	-	-
Jo 2023 [26]	1	B	N	MDA-MB-231 Cells	IP	Metastatic Breast Adenocarcinoma	EGFR	No, but TIGIT targeting RNP loaded RPs with CAR transgene	-	1 × 10^7^	3, 5	IP	Mock CAR-NK	26	Biol	-	0.2227	-	-
Xia 2023 [29]	1	A	N	EMT6-hHER2 Cells	SC	Murine Mammary Adenocarcinoma	HER 2	-	-	6 × 10^6^	7, 14, 21, 28 and 35	IV	PBS	28	Tumor Volume	0.6078	0.7197	-	-
Xia 2023 [29]	1	B	N	EMT6-hHER2 Cells	SC	Murine Mammary Adenocarcinoma	HER 2	PD-1 ECD	-	6 × 10^6^	7, 14, 21, 28 and 35	IV	PBS	28	Tumor Volume	0.1555	0.1841	-	-
Xia 2023 [29]	2	A	N	EMT6-hHER2 Cells	SC	Murine Mammary Adenocarcinoma	HER 2	-	-	6 × 10^6^	7, 14 and 21	IV	PBS	14	Tumor Volume	0.5326	0.6533	-	-
Xia 2023 [29]	2	B	N	EMT6-hHER2 Cells	SC	Murine Mammary Adenocarcinoma	HER 2	PD-1 ECD	sPD-1, 2.3 ng/nl, IP	6 × 10^6^	7, 14 and 21	IV	PBS	14	Tumor Volume	0.2609	0.3200	-	-
Xia 2023 [29]	2	C	N	EMT6-hHER2 Cells	SC	Murine Mammary Adenocarcinoma	HER 2	PD-1 ECD	-	6 × 10^6^	7, 14 and 21	IV	PBS	14	Tumor Volume	0.0870	0.1067	-	-
Xia 2023 [29]	3	A	PBMCs	JIMT-1 Cells	SC	Invasive Ductal Breast Carcinoma	HER 2	-	-	1 × 10^7^	7, 14	IV	PBS	7	Tumor Volume	0.3227	0.6806	-	-
Xia 2023 [29]	3	B	N	JIMT-1 Cells	SC	Invasive Ductal Breast Carcinoma	HER 2	PD-1 ECD	-	1 × 10^7^	7, 14	IV	PBS	7	Tumor Volume	0.2437	0.5139	-	-
Yang 2023 [6]	1	A	N	MD231 Cells	SC	Metastatic Breast Adenocarcinoma	MSLN	-	-	ns	Weekly dose × 7 Weeks	IV	Saline	42	Tumor Weight	0.4402	0.5468	-	-
Lee 2025 [30]	1	A	N	SK-BR-3 Cells	SC	Metastatic Breast Adenocarcinoma	CD19	IL-15	-	5 × 10^6^	5	IV	PBS	16	Tumor Volume	0.6210	-	-	-
Lee 2025 [30]	1	B	N	SK-BR-3 Cells	SC	Metastatic Breast Adenocarcinoma	HER3	IL-15	-	5 × 10^6^	5	IV	PBS	16	Tumor Volume	0.3508	-	-	-
Liu 2024 [11]	1	A	N	MDA-MB-468 Cells	Orthotopic	Metastatic Breast Adenocarcinoma	HER1	-	-	5 × 10^6^	16, 18	IV	Mock CAR-NK	14	Tumor Volume	-	0.5588	-	1.5200
Liu 2024 [11]	1	B	Puerarin@PEGel	MDA-MB-468 Cells	Orthotopic	Metastatic Breast Adenocarcinoma	HER1	-	-	5 × 10^6^	16, 18	IV	Mock CAR-NK	14	Tumor Volume	-	0.1059	-	2.8000
Rafei 2025 [31]	1	A	I	BCX.010 Cells	IV	-	CD70	IL-15	-	3 × 10^5^	7	IV	Untreated	35	No. of metastatic nodules	0.6714	0.4926	0.9700	0.7760
Rafei 2025 [31]	1	B	I	BCX.010 Cells	IV	-	CD70	CREM KO, IL-15	-	3 × 10^5^	7	IV	Untreated	35	No. of metastatic nodules	0.2827	0.2074	1.4400	1.1520
Gergely 2025 [5]	1	A	N	JIMT-1 Cells	SC	Invasive Ductal Breast Carcinoma	HER2	CD16 (FcγRIIIA 176V). Different CAR domains (CD28 and 41BB)	IL-2, 50,000 IU i.p., twice weekly	5 × 10^6^	14	IV	Unmodified NK Cells	21	Biol	-	0.7200	-	1.1304
Gergely 2025 [5]	1	B	N	JIMT-1 Cells	SC	Invasive Ductal Breast Carcinoma	HER2	CD16 (FcγRIIIA 176V), Different CAR domain (41BB)	IL-2, 50,000 IU i.p., twice weekly	5 × 10^6^	14	IV	Unmodified NK Cells	21	Biol	-	0.7173	-	1.1304
Gergely 2025 [5]	1	C	N	JIMT-1 Cells	SC	Invasive Ductal Breast Carcinoma	HER2	CD16 (FcγRIIIA 176V), Different CAR Domain (CD28)	IL-2, 50,000 IU i.p., twice weekly	5 × 10^6^	14	IV	Unmodified NK Cells	21	Biol	-	0.7707	-	1.1087
Liu 2025 [10]	1	A	N	MDA-MB-231 Cells	SC	Metastatic Breast Adenocarcinoma	FITC Folate	-	CAR hPSC-neutrophils (5.00 × 10^5^) FITC-FA (500 nmol)/kg every 3 days	5 × 10^5^	7	IV	PBS	14	Tumor Weight	0.0763	0.0789	-	-
Liu 2025 [10]	1	B	N	MDA-MB-231 Cells	SC	Metastatic Breast Adenocarcinoma	FITC Folate	-	FITC-FA (500 nmol)/kg every 3 days	5 × 10^5^	7	IV	PBS	14	Tumor Weight	0.6208	0.6425	-	-
Röder 2025 [1]	1	A	N	Organoid Cells	SC	-	HER2	-	-	1 × 10^7^	6, 9, 12, and 15	Peritumoral	Medium	12	Tumor Weight	0.5567	0.6408	-	-

**N**, no; **ROM_1/MSR_1**, CAR-NK vs True Control; **ROM_2/MSR_2**, CAR-NK vs Unmodified/Mock NK; **EGFR**, Epidermal Growth Factor Receptor; **oHSV-1**, Oncolytic Herpes Simplex Virus 1; **DPBS,** Dulbecco’s Phosphate-Buffered Saline; **HLA-G**, Human Leukocyte Antigen G; **iC9**, Inducible Caspase 9, a suicide gene; **DAP-12**, DNAX Activation Protein of 12 kDa; **IL-2/IL-15**, Interleukin-2/Interleukin-15; **PDX**, Patient-Derived Xenograft; **ScFv**, Single Chain Variable Fragment; **IP**, Intraperitoneal; **IV**, Intravenous; **TIGIT**, T-cell Immunoreceptor with Ig and ITIM domains; **RNP**, Ribonucleoprotein; **RPs**, Retroviral particles; **SC**, Subcutaneous; **PBS**, Phosphate-Buffered Saline; **HER1/HER2/HER3**, Human Epidermal Growth Factor Receptor 1/2/3; **PD-1**, Programmed Cell Death Protein 1; **ECD**, Extracellular Domain; **MSLN**, Mesothelin; **CREM**, cAMP Response Element Modulator; **CD16 (FcγRIIIA 176V)**, Cluster of Differentiation 16 (Fc gamma Receptor IIIA 176V); and **FITC-FA**, Fluorescein Isothiocyanate—Folic Acid.

**Table 3:** Subgroup analysis and multi-level meta-analysis to explore sources of heterogeneity and the impact of design variations.

3.1 Outcome: Ratio of Means (ROMs) Against True Control						3.2 Outcome: Median Survival Ratio (MSR) Against Mock Control					
Subgroup	*N* (Treatment Groups)	ROM (95% CI)	*p* (Within Group)	*p* (Between Groups)	I^2^ (%)	Subgroup	*N*	MSR (95% CI)	*p* (Within Group)	*p* (Between Groups)	*p* (Heterogeneity)
**Cell Source**						**Cell Source**					
PB	7	0.266 (0.142, 0.497)	0.002	0.181	97.355	PB	6	1.600 (1.230, 2.081)	0.006	0.063	0.68
NK-92	4	0.324 (0.122, 0.861)	0.035		84.010	NK-92	5	1.242 (0.969, 1.593)	0.073		0.78
CB	4	0.465 (0.260, 0.830)	0.025		80.026						
**Costimulatory Domain**						**IL Support**					
CD28	12	0.294 (0.194, 0.447)	<0.001	0.688	95.43	Yes	7	1.176 (0.937, 1.476)	0.132	**0.028**	0.52
4-1BB	4	0.349 (0.121, 1.003)	0.050		98.79	No	6	1.639 (1.205, 2.227)	0.009		0.67
**IL Support**											
Yes	4	0.465 (0.260, 0.830)	0.025	0.062	80.02	**Dose Variations**					
No	14	0.285 (0.189, 0.428)	<0.001		97.43	<5 M	4	1.132 (0.639, 2.009)	0.539	0.465	0.22
**Dose Variations**						5–10 M	7	1.323 (1.001, 1.748)	0.049		0.49
<5 M	6	0.275 (0.107, 0.706)	0.017	0.722	91.37	**Dose Frequency**					
5–10 M	11	0.318 (0.210, 0.482)	<0.001		96.75	Single	7	1.128 (0.899, 1.415)	0.241	**0.011**	0.625
**Dose Frequency**						Multiple	6	1.600 (1.230, 2.081)	0.006		0.679
Single	8	0.321 (0.164, 0.627)	0.005	0.875	97.0						
Multiple	10	0.304 (0.195, 0.473)	<0.001		96.50						
**3.3 Multi-Level Meta-Analysis with Rom to Further Explore Heterogeneity**											
	CAR-NK Vs. True Controls		CAR-NK Vs. Unmodified/mock NK Cells								
	σ^2^	I^2^	σ^2^	I^2^							
Study level	1.769 × 10^−10^	0.000	0.044	9.034							
Experiment level	3.524 × 10^−11^	0.000	7.018 × 10^−10^	0.000							
Treatment group level	0.416	93.040	0.384	78.840							

Abbreviations: **PB**, peripheral blood; **NK-92**, NK-92 and NK-92MI cell lines; **CB**, cord blood; **IL**, Interleukin (IL-2/IL-15); **5 M or 10 M**, 5 million cells or 10 million cells; **CI**, confidence interval; **σ^2^**, sigma square. Bold numerical values indicate statistically significant *p*-values. For MSR, unmodified/mock NK cells were chosen as the control due to an insufficient number of cases in the true control.

**Table 4:** Characteristics of clinical trials evaluating the safety and efficacy of CAR-NK cells in breast cancer.

No.	Trial ID (Clinicaltrials.gov and ICTRP)	Target Antigen	Target Conditions	Histopathological Characteristics of Breast Cancer	Source	CAR-NK Intervention Details	Trial Phase	First Posted	Trial Status	Expected Enrollment	Trial Sponsor	Country	Results Available
1	NCT07410494	Multiple	Breast Cancer, NSCLC, Colorectal Cancer (Locally Advanced or Metastatic), Prostate Cancer—Recurrent, Pancreatic Ductal Adenocarcinoma (PDAC), Ovarian Cancer, Glioblastoma, Melanoma (Skin Cancer), Acute Myeloid Leukemia (AML), Non-Hodgkin Lymphoma, Multiple Myeloma (MM), Lymphoma, Large B-Cell, Diffuse (DLBCL), Lymphoma, Liver Cancer	Advanced/unresectable or metastatic breast carcinoma	PB	Arm-1: Single-target CAR-NK cells Lymphodepleting therapy with CTX and FLU prior to CAR-NK Arm-2: Dual-target CAR-NK cells Same regimen as in arm-1 except dual-targeting CAR-NK cells are selected	Phase 1/2	13 February 2026	Recruiting	85	Essen Biotech	US	No
2	NCT07410676	Undisclosed	Sarcoma, Leukemia, Breast Cancer, Lung Cancer, Colorectal Cancer, Melanoma (Skin Cancer), Bladder Cancer, Kidney Cancer, Pancreatic Cancer Metastatic, Liver Cancer (Primary and Metastatic), Ovarian Cancer, Esophageal Cancer, Glioblastoma, Non-Melanoma Skin Cancer	Locally advanced or metastatic	Undisclosed	Lymphodepleting therapy with CTX and FLU followed by CAR-NK, low-dose IL-15 and Pembrolizumab	Phase 1/2	13 February 2026	Recruiting	83	Essen Biotech	US	No
3	IRCT20240610062069N1	EGFRvIII	Metastatic Breast Cancer	Metastatic	Undisclosed	Lymphodepleting therapy with CTX and FLU followed by CAR-NK	Phase 1	25 July 2024	Recruiting	6	Omid Cell and Tissue Processing Company	Iran	No
4	NCT06066424	TROP2	Non-Small Cell Lung Cancer (NSCLC), Breast Cancer	Locally advanced or metastatic HER2-negative/HER2-low breast cancer	Cord Blood	Lymphodepleting therapy with CTX and FLU followed by CAR-NK. Rimiducid to manage CAR-NK toxicity	Phase 1	4 October 2023	Recruiting	54	M.D. Anderson Cancer Center	US	No
5	NCT05686720	Undisclosed	TNBC	Advanced TNBC	Undisclosed	CAR-NK administered in escalating doses	Early Phase 1	1 February 2023	Not yet recruiting	12	First Affiliated Hospital of Shantou University Medical College	China	No
6	NCT05528341	NKG2DL	Breast Cancer, Lung Cancer, Gastric Cancer, Ovarian Cancer, Cervical Cancer, Renal Carcinoma, Malignant Melanoma, Osteosarcoma and Lymphoma	ns	NK-92	CAR-NK	Phase 1	26 January 2023	Recruiting	20	Xinxiang Medical University	China	No
7	NCT05678205	HER2	Breast Cancer, Gastric Cancer, Gastroesophageal Junction Adenocarcinoma	Advanced/unresectable or metastatic breast cancer with HER2 overexpression	Undisclosed	Lymphodepleting therapy with CTX and FLU followed by CAR-NK	Phase 1/2	1 January 2023	Not yet recruiting	133	Artiva Biotherapeutics	US	No
8	NCT05395052	MICA/B	Non-Small Cell Lung Cancer, Colorectal Cancer, Breast Cancer, Ovarian Cancer, Pancreatic Cancer, Head and Neck Squamous Cell Carcinoma, Gastroesophageal Junction Adenocarcinoma	Metastatic TNBC	iPSC	Lymphodepleting therapy with CTX and FLU followed by CAR-NK as a monotherapy or in combination with mAbs. IL-2 to maintain persistence	Phase 1	31 May 2022	Terminated	5	Fate Therapeutics	US	No
9	NCT04927884	PD-L1	TNBC	Advanced TNBC	NK-92	CAR-NK in combination with Sacituzumab, CTX, and IL-15 agonist	Phase 1/2	27 September 2021	Terminated	3	ImmunityBio, Inc	US	No
10	NCT05137275	5T4	NSCLC, TNBC, Colorectal Cancer, Mesothelioma	Advanced TNBC	Undisclosed	CAR-NK only	Early Phase 1	24 November 2021	Recruiting	56	Shanghai East Hospital	China	No
11	NCT02839954	MUC1	Hepatocellular Carcinoma, Non-Small Cell Lung Cancer, Pancreatic Cancer, Triple-Negative Breast Cancer, Glioblastoma, Colorectal Cancer, Gastric Cancer	Advanced triple-negative basal-like breast carcinoma	NK-92	CAR-NK only	Phase 1/2	1 July 2016	Unknown	10	PersonGen BioTherapeutics (Suzhou) Co., Ltd.	China	Yes (not for breast cancer)

## Data Availability

The original contributions presented in this study are included in the article/Supplementary Materials. Further inquiries can be directed to the corresponding authors.

## Supplementary Materials

The following supporting information can be downloaded at: https://www.mdpi.com/article/10.3390/cancers18101634/s1, Supplementary Figure S1: Funnel plots; Supplementary Table S1: PRISMA checklist; Supplementary Table S2: Search queries; Supplementary Table S3: Leave-one-out analysis on included estimates in Figure 2A,B; Supplementary Table S4: Safety information extracted from included studies; Supplementary Table S5: Persistence data extracted from included studies; Supplementary Table S6: Risk-of-bias assessments for each included study using SYRCLE’s risk-of-bias tool for animal studies; Supplementary Table S7: Trim-and-fill analyses of corresponding funnel plots; Supplementary Table S8: Clinical outcome data of ‘natural killer’ cell therapy in breast cancer extracted from Park et al. 2024 [35]. References [35,36,37,38,39] are cited in the Supplementary Materials.
