# Supplementary material for: Evaluation of Efficacy and Safety of Chimeric Antigen Receptor-Natural Killer (CAR-NK) Cells in Breast Cancer: A Systematic Review and Meta-Analysis
Source: Cancers (Basel). 2026 May 19;18(10):1634. doi: 10.3390/cancers18101634 (PMC13204228; doi:10.3390/cancers18101634)
Supplement: Supplementary file 1 [file cancers-18-01634-s001.zip › cancers-4241558-supplementary.pdf]

Supplementary Data

Supplementary Figure S1. Funnel Plots

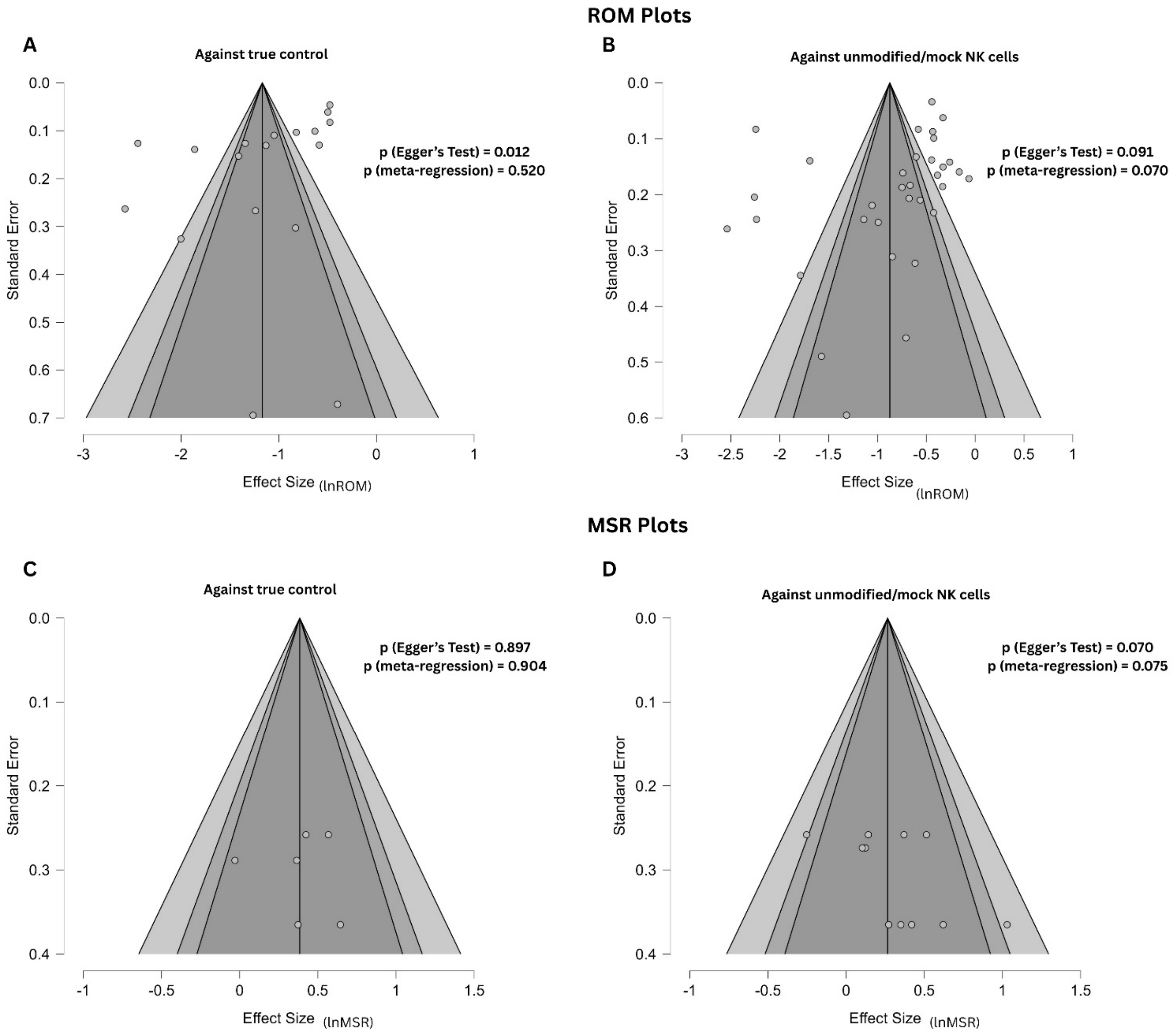

Supplementary Table S1. PRISMA checklist

Section and Topic	Item #	Checklist item	Page No./location where reported
**TITLE**			
Title	1	Identify the report as a systematic review.	1
**ABSTRACT**			
Abstract	2	See the PRISMA 2020 for Abstracts checklist.	2
**INTRODUCTION**			
Rationale	3	Describe the rationale for the review in the context of existing knowledge.	3, 4
Objectives	4	Provide an explicit statement of the objective(s) or question(s) the review addresses.	3, 4
**METHODS**			
Eligibility criteria	5	Specify the inclusion and exclusion criteria for the review and how studies were grouped for the syntheses.	Heading 2.3
Information sources	6	Specify all databases, registers, websites, organisations, reference lists and other sources searched or consulted to identify studies. Specify the date when each source was last searched or consulted.	Heading 2.2
Search strategy	7	Present the full search strategies for all databases, registers and websites, including any filters and limits used.	Table S2
Selection process	8	Specify the methods used to decide whether a study met the inclusion criteria of the review, including how many reviewers screened each record and each report retrieved, whether they worked independently, and if applicable, details of automation tools used in the process.	Heading 2.4
Data collection process	9	Specify the methods used to collect data from reports, including how many reviewers collected data from each report, whether they worked independently, any processes for obtaining or confirming data from study investigators, and if applicable, details of automation tools used in the process.	Heading 2.5
Data items	10a	List and define all outcomes for which data were sought. Specify whether all results that were compatible with each outcome domain in each study were sought (e.g. for all measures, time points, analyses), and if not, the methods used to decide which results to collect.	Heading 2.5
	10b	List and define all other variables for which data were sought (e.g. participant and intervention characteristics, funding sources). Describe any assumptions made about any missing or unclear information.	Heading 2.5
Study risk of bias assessment	11	Specify the methods used to assess risk of bias in the included studies, including details of the tool(s) used, how many reviewers assessed each study and whether they worked independently, and if applicable, details of automation tools used in the process.	Heading 2.7
Effect measures	12	Specify for each outcome the effect measure(s) (e.g. risk ratio, mean difference) used in the synthesis or presentation of results.	Heading 2.6
Synthesis methods	13a	Describe the processes used to decide which studies were eligible for each synthesis (e.g. tabulating the study intervention characteristics and comparing against the planned groups for each synthesis (item #5)).	Heading 2.5
	13b	Describe any methods required to prepare the data for presentation or synthesis, such as handling of missing summary statistics, or data conversions.	Heading 2.5, 2.6
	13c	Describe any methods used to tabulate or visually display results of individual studies and syntheses.	Heading 2.5
	13d	Describe any methods used to synthesize results and provide a rationale for the choice(s). If meta-analysis was performed, describe the model(s), method(s) to identify the presence and extent of statistical heterogeneity, and software package(s) used.	Heading 2.6
	13e	Describe any methods used to explore possible causes of heterogeneity among study results (e.g. subgroup analysis, meta-regression).	Heading 2.6
	13f	Describe any sensitivity analyses conducted to assess robustness of the synthesized results.	Heading 2.6, Figures 3, Supplementary Table S3
Reporting bias assessment	14	Describe any methods used to assess risk of bias due to missing results in a synthesis (arising from reporting biases).	Heading 2.6
Certainty assessment	15	Describe any methods used to assess certainty (or confidence) in the body of evidence for an outcome.	Heading 2.6
**RESULTS**			
Study selection	16a	Describe the results of the search and selection process, from the number of records identified in the search to the number of studies included in the review, ideally using a flow diagram.	Figure 1
	16b	Cite studies that might appear to meet the inclusion criteria, but which were excluded, and explain why they were excluded.	None

Section and Topic	Item #	Checklist item	Page No./location where reported
Study characteristics	17	Cite each included study and present its characteristics.	Table 1, Table 4
Risk of bias in studies	18	Present assessments of risk of bias for each included study.	Table S6
Results of individual studies	19	For all outcomes, present, for each study: (a) summary statistics for each group (where appropriate) and (b) an effect estimate and its precision (e.g. confidence/credible interval), ideally using structured tables or plots.	Table 2
Results of syntheses	20a	For each synthesis, briefly summarise the characteristics and risk of bias among contributing studies.	Heading 3.1.1, 3.1.2, 3.1.5
	20b	Present results of all statistical syntheses conducted. If meta-analysis was done, present for each the summary estimate and its precision (e.g. confidence/credible interval) and measures of statistical heterogeneity. If comparing groups, describe the direction of the effect.	Figures 2-3
	20c	Present results of all investigations of possible causes of heterogeneity among study results.	Heading 3.1.3
	20d	Present results of all sensitivity analyses conducted to assess the robustness of the synthesized results.	Figure 2; Table S3
Reporting biases	21	Present assessments of risk of bias due to missing results (arising from reporting biases) for each synthesis assessed.	Figure S1, Table S7
Certainty of evidence	22	Present assessments of certainty (or confidence) in the body of evidence for each outcome assessed.	CIs reported for each estimate
**DISCUSSION**			
Discussion	23a	Provide a general interpretation of the results in the context of other evidence.	Pages 14-15
	23b	Discuss any limitations of the evidence included in the review.	Page 16-17
	23c	Discuss any limitations of the review processes used.	Page 16-17
	23d	Discuss implications of the results for practice, policy, and future research.	Page 17
**OTHER INFORMATION**			
Registration and protocol	24a	Provide registration information for the review, including register name and registration number, or state that the review was not registered.	Heading 2.1
	24b	Indicate where the review protocol can be accessed, or state that a protocol was not prepared.	Heading 2.1
	24c	Describe and explain any amendments to information provided at registration or in the protocol.	Heading 2.8
Support	25	Describe sources of financial or non-financial support for the review, and the role of the funders or sponsors in the review.	Page 18
Competing interests	26	Declare any competing interests of review authors.	Page 18
Availability of data, code and other materials	27	Report which of the following are publicly available and where they can be found: template data collection forms; data extracted from included studies; data used for all analyses; analytic code; any other materials used in the review.	Supplementary Figures and Tables; Study Tables and Figures

Supplementary Table S2. Search queries

**Query**	**Database**
CAR-NK Search	
"CAR NK Cell*" OR “CAR-NK” OR "CAR Natural Killer Cells" OR "chimeric antigen receptor natural killer cell"	PubMed
"CAR-NK"[TIAB] OR "CAR NK"[TIAB] OR "CAR Natural Killer Cell*"[TIAB] OR "chimeric antigen receptor natural killer cell*"[TIAB]	PubMed
(((TITLE_ABS:(CAR NK Cell) AND ((SRC:(MED OR PMC OR AGR OR CBA) NOT PUB_TYPE:(Review)) OR PUB_TYPE:(Clinical Trial))) OR TITLE_ABS:(CAR natural killer Cell)) AND ((SRC:(MED OR PMC OR AGR OR CBA) NOT PUB_TYPE:(Review)) OR PUB_TYPE:(Clinical Trial)))	Europe PMC
"CAR NK Cell" OR "CAR-NK Cell" OR "chimeric antigen receptor natural killer cell" AND (LIMIT-TO (DOCTYPE , "ar")) AND (LIMIT-TO (LANGUAGE , "English")) AND (LIMIT-TO (SRCTYPE , "j"))	Scopus
(TS=(CAR NK Cell)) OR TS=(CAR-NK Cell)	WOS
((TS=(CAR NK)) OR TS=(CAR-NK)) OR TS=("Chimeric antigen receptor natural killer")	WOS
(CAR-NK cell):ti,ab,kw	Cochrane
(CAR NK):ti,ab,kw	Cochrane
(chimeric antigen receptor NK):ti,ab,kw	Cochrane
(chimeric antigen receptor natural killer cells):ti,ab,kw	Cochrane
CAR NK	ScienceDirect
**Clinical Trial Search**	
‘CAR-NK’	Clinicaltrials.gov
‘NK’ in Title; ‘Breast Cancer’ in Condition; ‘CAR-NK’ in ‘Intervention’; Timeframe, up to 01 March 2026	WHO International Clinical Trials Registry Platform (ICTRP)
Title and condition unspecified; ‘CAR-NK’ in ‘Intervention’; Timeframe, up to 01 March 2026	WHO International Clinical Trials Registry Platform (ICTRP)

Supplementary Table S3. Leave-one-out analysis on included estimates in Figure 2A,B.

Casewise Diagnostics Table for Figure 2A

	Leave One Out									
**Label**	Standardized Residual	DFFITS	Cook's Distance	Covariance ratio	τ	τ^2	Q_e	Hat	Weight	Influential
**Chen 2016-1A**	0.488	0.116	0.014	1.104	0.660	0.436	429.3	0.051	5.149	
**Chen 2016-1B**	-1.214	-0.279	0.076	1.024	0.637	0.405	415.5	0.050	5.007	
**Kim 2019-1A**	-0.101	-0.021	4.789×10^{-4}	1.123	0.665	0.442	426.5	0.054	5.365	
**Xia 2023-1A**	1.090	0.281	0.078	1.055	0.641	0.412	400.5	0.062	6.229	
**Xia 2023-1B**	-1.106	-0.281	0.078	1.050	0.640	0.409	368.7	0.060	6.006	
**Xia 2023-2A**	0.856	0.220	0.049	1.082	0.652	0.425	426.4	0.061	6.134	
**Xia 2023-2B**	-0.270	-0.065	0.005	1.126	0.666	0.444	409.8	0.061	6.052	
**Xia 2023-2C**	-2.292	-0.609	0.292	0.851	0.563	0.317	252.8	0.061	6.052	
**Xia 2023-3A**	0.058	0.018	3.374×10^{-4}	1.131	0.668	0.446	422.5	0.060	6.037	
**Xia 2023-3B**	-0.373	-0.091	0.009	1.120	0.664	0.441	412.7	0.059	5.950	
**Yang 2023-1A**	0.546	0.141	0.021	1.111	0.661	0.437	429.2	0.061	6.127	
**Lee 2025-1A**	1.123	0.288	0.082	1.050	0.640	0.410	412.7	0.062	6.183	
**Lee 2025-1B**	0.188	0.051	0.003	1.129	0.667	0.445	423.8	0.061	6.108	
**Rafei 2025-1A**	0.847	0.151	0.023	1.049	0.649	0.421	428.9	0.030	3.013	
**Rafei 2025-1B**	-0.101	-0.016	2.714×10^{-4}	1.094	0.656	0.430	428.8	0.029	2.908	
**Liu 2025-1A**	-2.354	-0.569	0.259	0.832	0.573	0.328	383.3	0.054	5.387	
**Liu 2025-1B**	1.130	0.291	0.083	1.050	0.640	0.409	360.9	0.063	6.253	
**Roder 2025-1A**	0.922	0.234	0.055	1.074	0.649	0.421	426.5	0.060	6.039	

Casewise Diagnostics Table for Figure 2B

Leave One Out

**Label**	Standardized Residual	DFFITS	Cook's Distance	Covariance ratio	τ	τ^2	Q_e	Hat	Weight	Influential
**Chen 2016-1A**	0.361	0.065	0.004	1.057	0.658	0.433	723.0	0.027	2.721	
**Chen 2016-1B**	-1.282	-0.214	0.045	1.007	0.642	0.412	712.0	0.026	2.649	
**Kim 2019-1A**	0.462	0.087	0.008	1.058	0.658	0.433	722.8	0.031	3.073	
**Hu 2020-1A**	-2.209	-0.417	0.155	0.920	0.608	0.369	660.2	0.031	3.088	
**Liu 2020-1A**	0.696	0.132	0.018	1.051	0.656	0.430	717.7	0.033	3.318	
**Liu 2020-1B**	0.682	0.130	0.017	1.052	0.656	0.430	716.8	0.033	3.334	
**Liu 2020-2A**	0.202	0.042	0.002	1.065	0.660	0.436	722.7	0.032	3.197	
**Liu 2020-2B**	0.189	0.039	0.002	1.064	0.660	0.436	722.7	0.031	3.134	
**Liu 2020-3A**	-0.266	-0.042	0.002	1.062	0.659	0.435	719.5	0.030	3.047	
**Liu 2020-3B**	-0.171	-0.025	6.403×10^{-4}	1.063	0.659	0.435	721.1	0.030	2.957	
**Liu 2020-4A**	1.091	0.198	0.039	1.027	0.648	0.419	713.7	0.032	3.202	
**Liu 2020-4B**	1.246	0.223	0.049	1.015	0.644	0.414	711.3	0.032	3.173	
**Liu 2020-5A**	0.037	0.011	1.278×10^{-4}	1.062	0.659	0.435	722.6	0.028	2.760	
**Liu 2020-5B**	0.295	0.058	0.003	1.062	0.660	0.435	723.0	0.031	3.082	
**Jo 2023-1A**	-0.507	-0.068	0.005	1.043	0.654	0.427	721.7	0.018	1.843	
**Xia 2023-1A**	0.852	0.161	0.026	1.044	0.653	0.426	694.2	0.034	3.364	
**Xia 2023-1B**	-1.276	-0.238	0.055	1.014	0.641	0.410	665.6	0.032	3.245	
**Xia 2023-2A**	0.659	0.119	0.014	1.050	0.656	0.430	722.1	0.030	3.009	
**Xia 2023-2B**	-0.386	-0.063	0.004	1.059	0.658	0.433	718.9	0.030	2.973	

**Xia 2023-2C**	-2.117	-0.388	0.137	0.929	0.613	0.376	680.3	0.030	2.973
**Xia 2023-3A**	0.742	0.137	0.019	1.048	0.655	0.429	720.5	0.032	3.188
**Xia 2023-3B**	0.311	0.061	0.004	1.062	0.660	0.435	723.0	0.031	3.144
**Yang 2023-1A**	0.412	0.080	0.007	1.061	0.659	0.435	722.9	0.033	3.259
**Liu 2024-1A**	0.451	0.088	0.008	1.061	0.659	0.434	722.4	0.033	3.340
**Liu 2024-1B**	-2.309	-0.463	0.187	0.911	0.598	0.358	329.4	0.033	3.340
**Rafei 2025-1A**	0.210	0.036	0.001	1.055	0.657	0.432	723.0	0.023	2.271
**Rafei 2025-1B**	-0.873	-0.129	0.017	1.030	0.650	0.423	719.4	0.022	2.164
**Gergely 2025-1A**	0.833	0.154	0.024	1.043	0.653	0.427	718.5	0.032	3.221
**Gergely 2025-1B**	0.816	0.149	0.022	1.043	0.653	0.427	720.1	0.031	3.138
**Gergely 2025-1C**	0.943	0.173	0.030	1.037	0.651	0.424	715.5	0.032	3.240
**Liu 2025-1A**	-2.644	-0.487	0.201	0.867	0.592	0.351	670.2	0.029	2.921
**Liu 2025-1B**	0.674	0.129	0.017	1.053	0.656	0.431	666.8	0.034	3.385
**Roder 2025-1A**	0.655	0.123	0.016	1.052	0.656	0.431	720.8	0.032	3.248

Supplementary Table S4. Safety information extracted from included studies.

**Chen 2016**	No comments
**Kim 2019**	No comments
**Jan 2021**	No comments
**Hu 2020**	Tissue factor (TF) targeted CAR-NK cells were not associated with weight loss.<br>Supporting quotes:<br>*"Net mouse body weights had no difference between control mice and treated mice (p=0.3314, for 18.6 grams of control mice vs. 19.1 grams of treated mice) (Figure 5C)."*<br>*"There was no statistical difference in net mouse body weights between control and NK-CAR1 treated groups (all p>0.05) (Fig. 4C), suggesting that TF-targeted CAR1-NK therapy was safe."*
**Liu 2020**	EGFR targeted CAR-NK cells treatment did not alter the mouse body weight.<br>Supporting quote:<br>*"The body weights of the mice were not affected by treatment with EGFR- CAR-1, EGFR-CAR-2, or Con-CAR NK cells in both xenograft models, suggesting that the tumor sizes were not affected by the health condition of the mice (Figure 4D,H,L,P and Figure 5F)."*
**Jo 2023**	No comments
**Xia 2023**	HER-2 targeting CAR-NK cells treatment with or without PD-1 addition did not alter mouse body weight as well as didn't display signs of cytokine toxicities. No major organ toxicities were observed.<br>Supporting quotes:<br>*"Mice in all groups injected with various NK cells did not exhibit any significant changes in body weight compared with the PBS control (Figure 3H)."*<br>*"[The authors measured 'temperature' as an indication of CRS] The results showed that the body temperature of the mice in each group were not altered compared to the PBS group (Figure 4F). Moreover, there was no significant change in mouse weight for all groups (Figure 4G)."*<br>*"There was no significant difference in body weight between the constructed-NK-cell-treated mice and the PBS control mice (Figure 5F). To discover if the sPD-1-CAR-NK cells impaired important organs in mice, an H&E staining assay was carried out.*

	Results showed that, similar to the PBS group, no significant changes were found in the heart, liver, spleen, lung and kidney tissue of mice after treatment with various NK cells (Figure 5H).”
**Yang 2023**	Mesothelin (MSLN) targeting CAR-NK cells were well tolerated.<br><br>Supporting Quote:<br><br>*“Importantly, the mice’s weights remained relatively stable throughout the experiment, indicating that the administration of MSLN-NK cells did not adversely affect their health (Fig. 4C).”*
**Lee 2025**	HER3 (ErbB3) did not implicate weigh loss in experimental mice.<br><br>Supporting quote:<br><br>*“aErbB3 CAR-NK cells exhibited a higher antitumor effect than aCD19 CAR-NK cells without significant side effects such as spinal curvature or weight loss (Supplementary Fig. S3).”*
**Liu 2024**	HER-1 targeting CAR-NK treatment didn’t disturb mice body weight.<br><br>Supporting quote:<br><br>*“In addition, such treatments minimally disturbed the body weights of treated mice (Figure S10B, Supporting Information).”*
**Gergely 2025**	No comments
**Rafei 2025**	CAR-NK treatment in mice directed against CD70 did not infer weight loss, organ toxicities, or abnormal renal/hematology profiles.<br><br>Supporting quote:<br><br>*“Treatment with CREM KO CAR70/IL-15 NK cells was well tolerated with no weight loss, organ toxicities, and stable renal/hematological profiles (Extended Data Fig 9h and Fig 10).”*
**Liu 2025**	Folate receptor targeting CAR-NK cells were well tolerated with no weight changes in mice or organ toxicities.<br><br>Supporting quote:<br><br>*"In addition, during the treatment period, the body weight of the mice changed relatively stable (Fig. 6H), and no significant damage occurred in the major organs."*
**Roder 2025**	No Comments

Supplementary Table S5. Persistence data extracted from included studies

Study ID	Exp ID	Compartment	Antigen	Additional variations	Total dose administered before measurement	Route	Measurement method	Day reported	Marker	Measurement	Measurement unit	Control NK	Control (Unit)	Last measured day	Diminishing day
**Jan 2021**	1A	Spleen	HLA-G	iC9, DAP-12<br><br>IL-15, 10ng/mouse, daily<br>IL-2, 10000U/mouse, q2d	1.50E+07	IV	Flowcytometry	7	CD56+	2070.588	Cells/12500 spleenocytes	Control absent		7	Not measured
**Jan 2021**	1B	Spleen	HLA-G	iC9, DAP-12<br><br>IL-15, 10ng/mouse, daily<br>IL-2, 10000U/mouse, q2d<br><br>AP20187 (iC9 inducer) also coadministered	1.50E+07	IV	Flowcytometry	7	CD56+	211.764	Cells/12500 spleenocytes	Control absent		7	Not reported

**Xia 2023**	1A	Tumor	HER2	-	1E7 x 2	IV	Flowcytometry	>= 14	CD56+	173	Cell count	78	Cell count	~14	Not reported
**Rafei 2025**	1A-i	Blood	CD70	IL-15 incorporation	1E6 or 3E6	IV	Flow Cytometry	10	CD16+CD70+	0.38	Cells/ul	0	Cells/ul	20	Not reported
**Rafei 2025**	1B-i	Blood	CD70	IL-15, CREM gene knock out	1E6 or 3E7	IV	Flow Cytometry	10	CD16+CD70+	0.61	Cells/ul	0	Cells/ul	20	Not reported
**Rafei 2025**	1A-ii	Blood	CD70	IL-15 incorporation	1E6 or 3E8	IV	Flow Cytometry	20	CD16+CD70+	1.11	Cells/ul	0.1	Cells/ul	20	Not reported
**Rafei 2025**	1B-ii	Blood	CD70	IL-15, CREM gene knock out	1E6 or 3E9	IV	Flow Cytometry	20	CD16+CD70+	3.15	Cells/ul	0.1	Cells/ul	20	Not reported
**Roder 2025**	1A	Tumor	HER2	-	1E7 x 4	Peritumoral	H&E	11	CD45+	Detected	-	Not detected	-	11	Not reported

Supplementary Table S6. Risk of bias assessments for each included study using SYRCLE's risk of bias tool for animal studies

No.	Study ID	Year	1-Sequence allocation		2-Balanced Baseline		3-Allocation Concealment		4-Random Housing		5-Blinding of Investigators		6-Random Outcome		7-Blinding (For outcome		8-Incomplete Data		9-Selective Outcome		10-Other sources	
			Tumor	Survival	Tumor	Survival	Tumor	Survival	Tumor	Survival	Tumor	Survival	Tumor	Survival	Tumor	Survival	Tumor	Survival	Tumor	Survival	Tumor	Survival
1	Chen 2016	2016	No	No	Yes	Yes	Unclear	Unclear	Unclear	Unclear	No	No	Unclear	N/A	Unclear	N/A	Yes	Yes	Yes	Yes	Yes	Yes
2	Kim 2019	2019	Unclear	N/A	Unclear	N/A	No	N/A	Unclear	N/A	No	N/A	Unclear	N/A	No	N/A	Yes	N/A	Yes	N/A	Yes	N/A
3	Jan 2021	2021	Unclear	Unclear	Unclear	Unclear	Unclear	Unclear	Unclear	Unclear	No	No	Unclear	N/A	Unclear	N/A	Probably Yes	Probably Yes	Yes	Yes	Yes	Yes
4	Hu 2020	2020	Unclear	Unclear	Unclear	Yes	Unclear	Unclear	Unclear	Unclear	Unclear	Unclear	Unclear	N/A	No	N/A	No	No	Probably Yes	Probably Yes	Yes	Yes
5	Liu 2020	2020	Unclear	Unclear	Yes	Yes	Unclear	Unclear	Unclear	Unclear	No	No	Unclear	N/A	No	N/A	Yes	Yes	Yes	Yes	Yes	Yes

6	Jo 2023	2023	Unclear	Unclear	Yes	Yes	No	No	Unclear	Unclear	No	No	Unclear	N/A	No	N/A	Yes	Yes	Yes	Yes	Yes	Yes
7	Xia 2023	2023	Unclear	Unclear	Yes	Yes	Unclear	Unclear	Unclear	Unclear	No	No	Unclear	N/A	No	N/A	Yes	Yes	Yes	Yes	Yes	Yes
8	Yang 2023	2023	No	N/A	Yes	N/A	Unclear	N/A	Unclear	N/A	No	N/A	Unclear	N/A	No	N/A	Yes	N/A	Yes	Yes	No	N/A
9	Lee 2025	2025	Unclear	Unclear	Yes	Yes	Unclear	Unclear	No	No	No	No	Unclear	N/A	No	N/A	Yes	Yes	Unclear	Unclear	No	No
10	Liu 2024	2024	Unclear	Unclear	Yes	Yes	Unclear	Unclear	Unclear	Unclear	No	No	Unclear	N/A	No	N/A	Yes	Yes	Yes	Yes	Yes	Yes
11	Rafei 2025	2025	Yes	Yes	Unclear	Unclear	Unclear	Unclear	Unclear	Unclear	Yes	Yes	Unclear	N/A	Yes	N/A	Yes	Yes	Unclear	Unclear	Yes	Yes
12	Gergely 2025	2025	No	No	Yes	Yes	Unclear	Unclear	Unclear	Unclear	No	No	Unclear	N/A	No	N/A	Yes	Yes	Yes	Yes	Yes	Yes
13	Liu 2025	2025	Unclear	Unclear	Yes	Yes	Unclear	Unclear	No	No	Unclear	Unclear	Unclear	N/A	No	N/A	Yes	Yes	Yes	Yes	Unclear	Unclear
14	Roder 2025	2025	No	No	Unclear	Unclear	No	No	Unclear	Unclear	No	No	Unclear	N/A	Unclear	N/A	Yes	Yes	Yes	Yes	Unclear	Yes

Supplementary Table S7: Trim and fill analyses of corresponding funnel plots

	**Trim and Fill**	**Adjusted Estimate μ**			
**Estimates**	**Missing Estimates**	**Estimate**	**Lower 95% CI**	**Upper 95% CI**	**p**
***Trim and Fill Parameter Estimates* for Figure S1 - A**					
18	0	-1.168	-1.485	-0.852	< .001
***Trim and Fill Parameter Estimates* for Figure S1 - B**					
33	0	-0.874	-1.108	-0.639	< .001
Trim and Fill Parameter Estimates for Figure S1 - C					
6	2	0.283	0.078	0.488	.007
***Trim and Fill Parameter Estimates* for Figure S1 - D**					
13	4	0.142	-0.004	0.287	.056

Supplementary Table S8. Clinical outcome data of 'natural-killer' cell therapy in breast cancer extracted from Park et al. 2024 [35]

Study ID (Year)	Patient number (*)	Clinical outcome	Age (years)	Gender M/(F)	Type of Cancer	Origin	Duration of expansion	Cytokines for expansion	Dose	Lymphodepleting chemotherapy	IL-2 administration	Combination therapy
**Lee, S. C. 2020**	31	PR1 SD16 PD14	32-73	–	Mixed advanced HER2-positive solid tumors refractory to standard therapy (breast ca. (30), gastric ca. (1))	Allo	–	–	1X10^6cells/kg,1X10^7 cells/kg, 5X10^7 cells/kg, 1X10^8 cells/kg X 3~19 infusions	No	Yes 1 million IU/m2	Trastuzumab with or without Bevacizumab
**Liang, S. 2017**	32	CR3 PR12 SD10 PD7	26-71	0 (18)	Breast ca.	Allo	12 days	No	–	No	No	Cryoablation with or without Herceptin.
**Liang, S. 2017**	36	PR4 SD22 PD10	around 50 yrs old	0 (36)	Breast ca.	Mixed	12 days	No	–	No	No	No
**Geller, M. A. 2011**	6	SD4 PD2	30-65	0 (20)	Mixed refractory metastatic breast ca. (6)	Allo	14 days	No	2.16X10^7 cells/kg (8.33X10^6 – 3.94X10^7)	Cy + Flu	Yes 10 MU	No
**Total Patients**	**105**											
**Complete Response**	3 (2.85%)											

**Partial Response**	17 (16.19%)
**Stable Disease**	52 (49.5%)
**Progressive Disease**	33 (31.4%)

References:

Geller, M. A., Cooley, S., Judson, P. L., Ghebre, R., Carson, L. F., Argenta, P. A., Jonson, A. L., Panoskaltsis-Mortari, A., Curtsinger, J., McKenna, D., Dusenbery, K., Bliss, R., Downs, L. S., & Miller, J. S. (2011). A phase II study of allogeneic natural killer cell therapy to treat patients with recurrent ovarian and breast cancer. *Cytotherapy*, *13*(1), 98–107. https://doi.org/10.3109/14653249.2010.515582

Lee, S. C., Shimasaki, N., Lim, J. S. J., Wong, A., Yadav, K., Yong, W. P., Tan, L. K., Koh, L. P., Poon, M. L. M., Tan, S. H., Ow, S. G. W., Bharwani, L., Yap, Y. S., Foo, M. Z. Q., Coustan-Smith, E., Sundar, R., Tan, H. L., Chong, W. Q., Kumarakulasinghe, N. B., Lieow, J. L. M., … Campana, D. (2020). Phase I Trial of Expanded, Activated Autologous NK-cell Infusions with Trastuzumab in Patients with HER2-positive Cancers. *Clinical cancer research : an official journal of the American Association for Cancer Research*, *26*(17), 4494–4502. https://doi.org/10.1158/1078-0432.CCR-20-0768

Liang, S., Xu, K., Niu, L., Wang, X., Liang, Y., Zhang, M., Chen, J., & Lin, M. (2017). Comparison of autogeneic and allogeneic natural killer cells immunotherapy on the clinical outcome of recurrent breast cancer. *OncoTargets and therapy*, *10*, 4273–4281. https://doi.org/10.2147/OTT.S139986

Liang, S., Niu, L., Xu, K., Wang, X., Liang, Y., Zhang, M., Chen, J., & Lin, M. (2017). Tumor cryoablation in combination with natural killer cells therapy and Herceptin in patients with HER2-overexpressing recurrent breast cancer. *Molecular immunology*, *92*, 45–53. https://doi.org/10.1016/j.molimm.2017.10.003

Park, H., Kim, G., Kim, N., Ha, S., & Yim, H. (2024). Efficacy and safety of natural killer cell therapy in patients with solid tumors: a systematic review and meta-analysis. *Frontiers in immunology*, *15*, 1454427. https://doi.org/10.3389/fimmu.2024.1454427
